# Supplementary material for: A 430 kyr record of ice-sheet dynamics and organic-carbon burial in the central Eurasian Arctic Ocean
Source: Nat Commun. 2025 Apr 23;16:3822. doi: 10.1038/s41467-025-59112-7 (PMC12019407; doi:10.1038/s41467-025-59112-7)
Supplement: Supplementary file 1 — Supplementary Information [file 41467_2025_59112_MOESM1_ESM.pdf]

# A 430 kyr record of ice-sheet dynamics and organic-carbon burial in the central Eurasian Arctic Ocean

Ruediger Stein[1,2,3]*, Thomas Frederichs[1], Kirsten Fahl[2], Walter Geibert[2], Jens Matthiessen[2], Frank Niessen[2], Christoph Vogt[1], Cynthia Sassenroth[2,4], Evgenia Bazhenova[1]

[1] Faculty of Geosciences and Center for Marine Environmental Sciences (MARUM), University of Bremen, Germany
[2]Alfred Wegener Institute (AWI) Helmholtz Centre for Polar and Marine Research, Bremerhaven, Germany
[3]Frontiers Science Center for Deep Ocean Multispheres and Earth System and Key Laboratory of Marine Chemistry Theory and Technology, Ocean University of China (OUC), Qingdao, China
[4]Present address: Department of Earth Sciences, University of Gothenburg, Göteborg, Sweden
* Corresponding author: ru_st@uni-bremen.de

## Supplementary Information (No. 1 to No. 7)

**(1) Supplementary Information on Polarstern Expedition PS115/2, coring/sampling, lithostratigraphy, and core correlation (Supplementary Figures 1 to 4)**
**(2) Supplementary information on the magnetic mineral composition, the degree of eventual oxidation, and spectral analysis of RPI records (Supplementary Figures 5 to 7)**
**(3) Supplementary information of correlation of RPI record with tuning target and alternative age model (Supplementary Figure 8)**
**(4) Supplementary information on organic carbon source and maturity (Supplementary Figures 9 to 10)**
**(5) Supplementary information on mineralogical proxy records, source regions and glacial deposits (Supplementary Figures 11 to 17)**
**(6) Supplementary Tables 1 to 4**
**(7) References**

**(1) Supplementary information on Polarstern Expedition PS115/2, coring/sampling, lithostratigraphy, and core correlation (Supplementary Figures 1 to 4)**

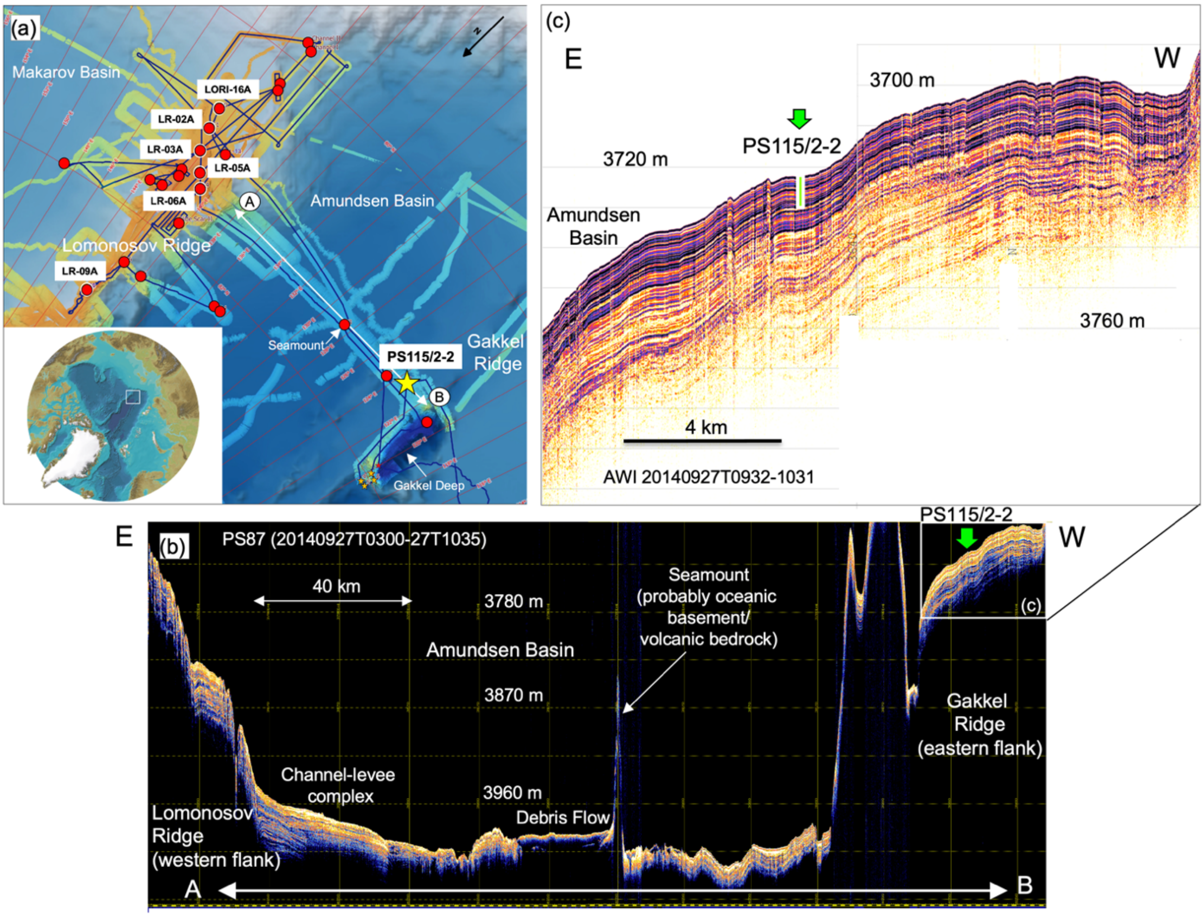


**Supplementary Fig. 1**
**Cruise track, bathymetric map and Parasound profile from Polarstern Expedition PS115/2**. (a) Cruise and hydrosweep track of Expedition PS115/2 and selected tracks from earlier Polarstern expeditions. PS115/2 geological stations are shown as red circles, location of Core PS115/2-2 is highlighted as large yellow star. Locations of potential future IODP drill holes, i.e., LR-09A, LR-06A, LR-05A, LR-03A, LR-02A and LORI-16A, are marked by white circles[1,2]. A-B marks location of Parasound profile of (b). The location of the study area of Expedition PS115/2 is shown as open white square in overview map in the left lower corner, representing the IBCAO International Bathymetric Chart of the Arctic Ocean (IBCAO)[3]. (b) Parasound profile crossing the Amundsen Basin from western flank of the Lomonosov Ridge to the eastern flank of the Gakkel Ridge and showing distinct topographic and sedimentary features such as prominent topographic highs, channel-levee complex, and debris flow, and the location of Core PS115/2-2. (c) Enlargement of part of Parasound profile across the PS115/2-2 core location showing a sequence of undisturbed stratified sediments. (Figure from ref. 1, supplemented).

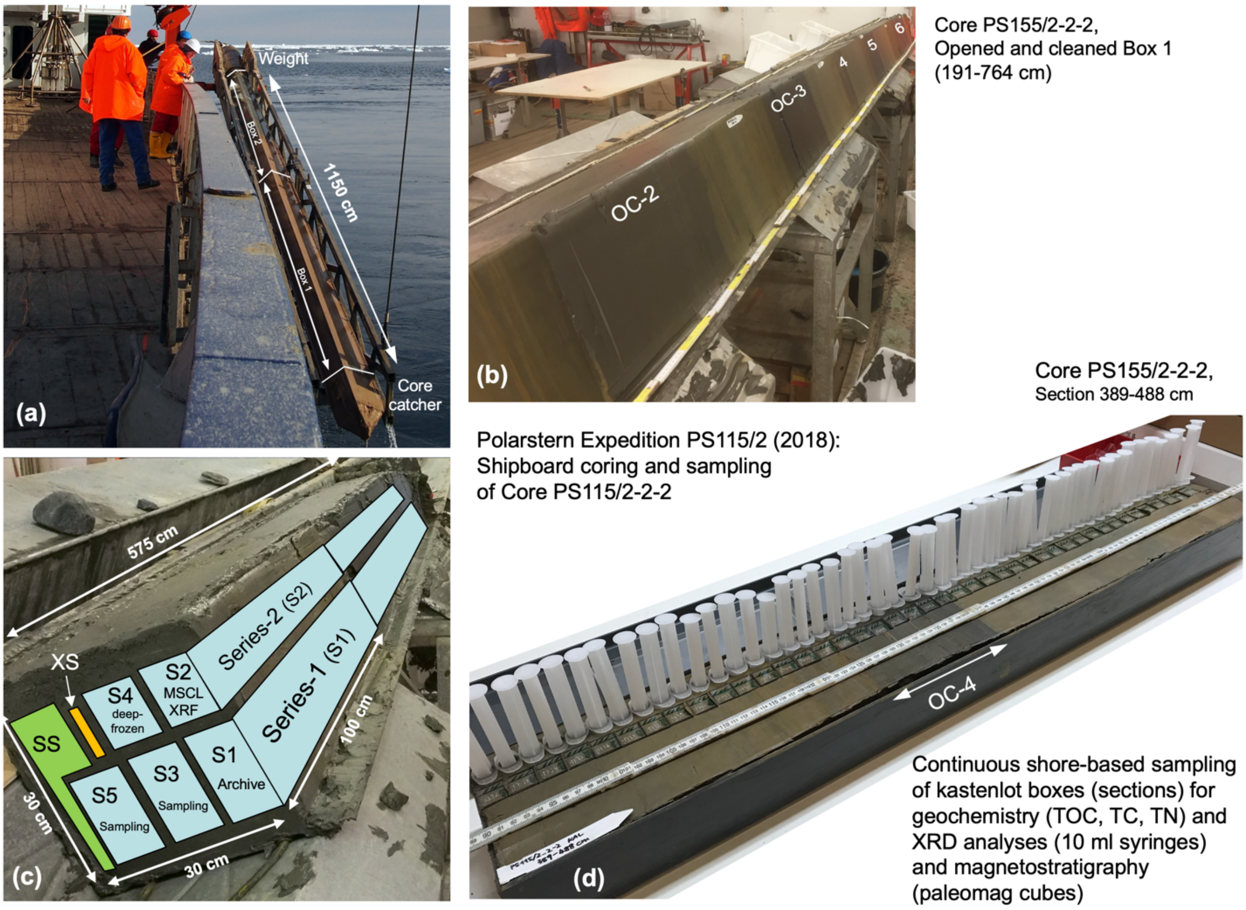


**Supplementary Fig. 2**

**Coring and sampling activities of Core PS115/2-2-2** (ref. 1). (a) Core PS115/2-2-2 was recovered by means of a “Kastenlot” Corer during Polarstern Expedition PS115/2 in 2018. (b) Opened and cleaned metal box with the lower sedimentary sequence of Core PS115/2-2-2. The well preserved dark gray intervals OC-2 to OC-6 are marked. (c) Schematic illustration of the onboard sampling process. A series of five sub-cores was taken in one-meter-long plastic boxes for different purposes (Archive, MSCL logging, XRF scanning; core description; sampling; etc., with one set of sub-cores that has been deep-frozen for later biomarker studies directly after the sampling); sediment slabs were taken for X-Ray photography (XS); finally, additional single samples (SS) were taken for other shipboard and/or shore-based analyses. (d) One series of Core PS115/2 has been selected for a continuous detailed sampling in the sediment lab of the Alfred Wegener Institute. As example the core section 389-488 cm (that contains the dark gray OC-4 interval) is shown. (Photographs R. Stein/AWI & University of Bremen).

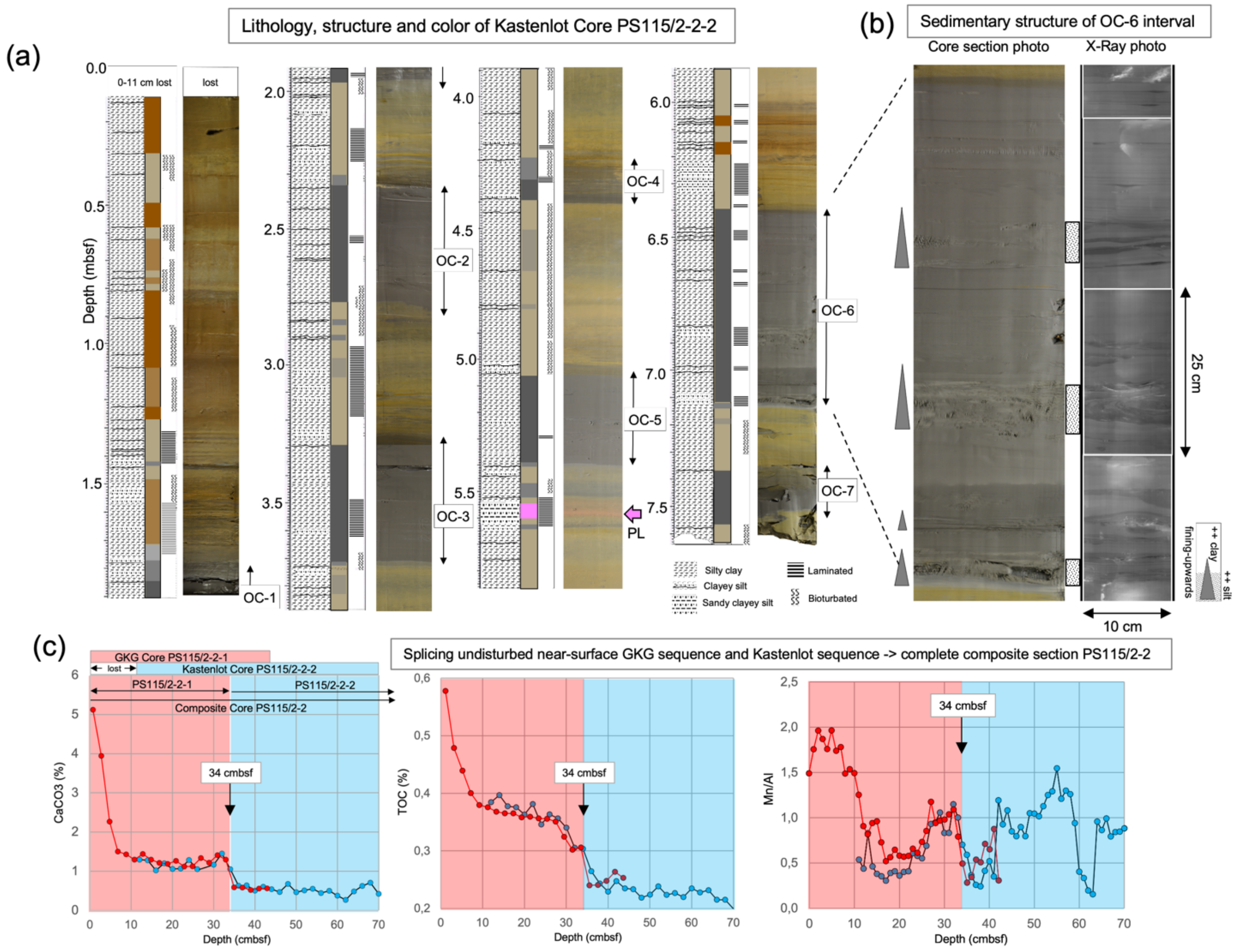


**Supplementary Fig. 3**

**Lithologies, sedimentary structures, and colors of Kastenlot Core PS115/2-2-2**. (a) Composite photograph and lithologies and sedimentary structures based on visual core description[1]. The dark gray OC-rich intervals OC-1 to OC-7 and the prominent "pink layer" (PL) are highlighted. (b) Blow-up of the OC-6 interval with core section photograph and X-Ray photograph, showing internal sedimentary structures in detail[1,4,5]. (c) Using selected geochemical data (i.e., $CaCO_3$ calculated as IC * 8.333 and TOC) and XRF-derived elemental ratios (e.g., Mn/Al), the near-surface sediments of box corer (GKG) Core PS115/2-2-1 have been combined with Kastenlot Core PS115/2-2-2 into one complete and undisturbed composite sedimentary sequence (spliced Core PS115/2-2) (This study). Source data of Supplementary Figure 3c are provided as a Source Data file.

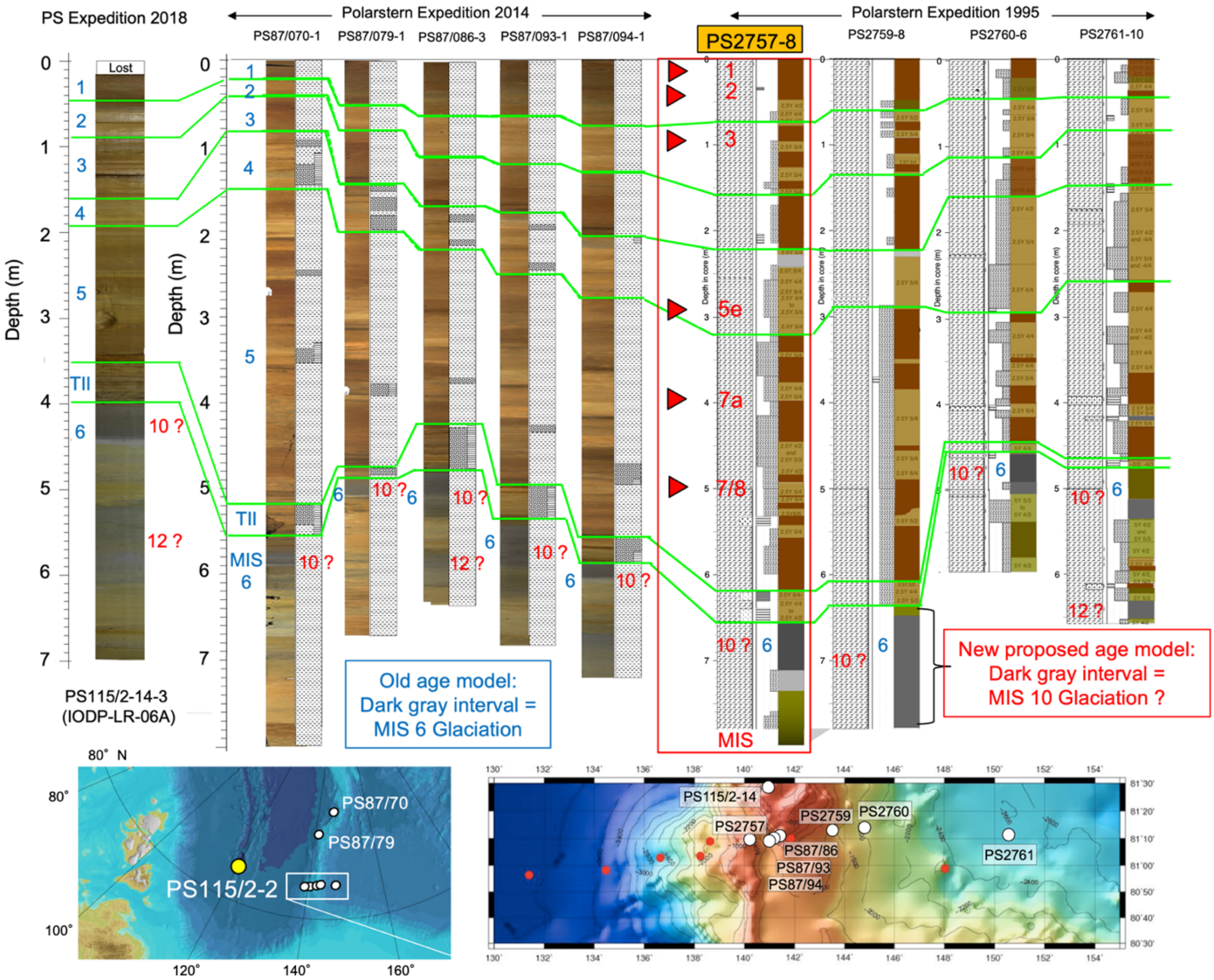


**Supplementary Fig. 4**

**Main lithologies and lithostratigraphy of selected sediment cores** (white circles on IBCAO-based bathymetric map[3]) recovered during Polarstern expeditions in 1995, 2014, and 2018 (refs. 1,6-8). Core PS115/2-14-3 has been recovered from the same location as the proposed IODP Primary Site LR-06A (LR-11A) (ref. 1, 2). The tentative age model (MIS 6 to 1) based on shipboard data and core correlation, is shown in blue letters[1,7-9]. Following this age model, the dark gray (OC-rich) sediments in the lowermost part of the sedimentary sections were interpreted as glacial deposits correlating with the MIS 6 glaciation. Based on new Optical Stimulated Luminescence (OSL) and $^{230}Th$ records, this tentative age model has been questioned in more recent studies[10,11]. In red letters, an alternative age model based on $^{230}Th$ data is shown for Core PS2757-8, indicating that the sediments at about 5 m core depth might have an MIS 7/8 age (see Supplementary Fig. 8f)[11]. Using this new age model would suggest that the dark gray sedimentary unit(s) recovered in all these cores, might represent glaciations older than MIS 6, i.e., MIS 10 or even MIS 12 (?). That means, these cores might represent a similar time interval as that of Core PS115/2-2. If this is correct, earlier reconstructions of the late Quaternary glacial history based on the old tentative age model have to be revised[9,12]. Furthermore, this also indicates that in the sedimentary sections shown here, not all glacial intervals (e.g., MIS 6) seem to be represented by dark gray OC-rich sediments (as it is the case for Core PS115/2-2), suggesting different source rocks/regions of the detrital sediments and/or differences in the ice sheet extent.

**(2) Supplementary information on the magnetic mineral composition, the degree of eventual oxidation, and spectral analysis of RPI records (Supplementary Figures 5 to 7)**

For the entire spliced Core PS115/2-2, rock magnetic properties together with the inclination and RPI records are shown in Supplementary Figure 5. To investigate whether and to what extent samples of normal and reverse magnetic polarity differ, we carried out temperature-dependent measurements of magnetic remanence on nine selected samples. These measurements are used to obtain information on the magnetic mineral composition and the degree of eventual oxidation. For this approach, we selected three samples with normal polarity and two samples with inverse polarity from the interval 445 - 490 cm core depth, used for temperature-dependent measurements on magnetic remanence (white circles). In addition, in order to elucidate to what extent the organic carbon (OC) content eventually has an influence on the magnetomineralogy, we took four samples from the interval 613 - 697 cm core depth (white circles), two each from areas that are of brownish color and OC-poor and of dark gray color and OC-rich, respectively:

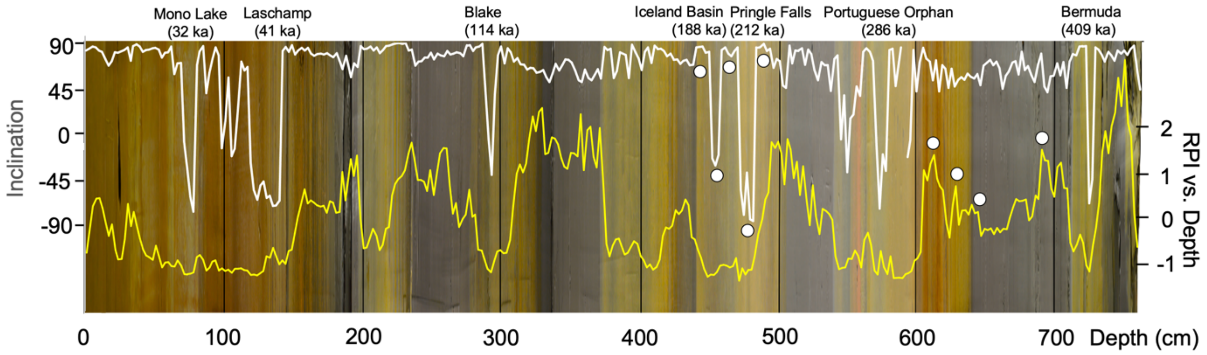


Furthermore, we added to each of the nine samples (listed as sample depth) a code that represents inclination and polarity of characteristic remanent magnetization and some sediment properties (color, organic carbon content, siderite):

| Depth (cm) | Code | Inclination (°) | Polarity |
|---|---|---|---|
| 445,4 | NbrOCp | 86,5 | normal |
| 454,7 | RbrOCp | -31,3 | reverse |
| 461,6 | NlgOCp | 85,5 | normal |
| 479,7 | RbrOCp | -84,4 | reverse |
| 489,6 | NbrOCp | 88,9 | normal |
| 613,7 | NbrOCp | 72,8 | normal |
| 629,6 | NbrOCp | 53,3 | normal |
| 645,7 | NdgOCr | 48,4 | normal |
| 696,3 | NdgOCrSid | 57 | normal |

**Explanation of abbreviations**

N, R: normal (reverse) polarity
br: brownish
lg, dg: light (dark) gray
OCp, OCr: organic-carbon poor (rich)
Sid: siderite

We measured the zero-field warming curves from 5 K to 300 K of a remanent magnetization acquired at a temperature of 5 K in a 5 T magnetic DC field after cooling in a zero field (**ZFC**) and secondly after cooling in a 5 T DC field (**FC**) (ref. 13). We also measured the cooling and rewarming curves of the remanent magnetization acquired in a 5 T DC field at 300 K between 300 K and 5 K and back to 300 K (RT (room temperature)-SIRM cycling)[14] (Supplementary Fig. 6).

In the **ZFC** measurements (Supplementary Fig. 6a and 6b) of samples 445.4 cm (NbrOCp), 454.7 cm (RbrOCp), 461.6 cm (NlgOCp) and 479.7 cm (RbrOCp), the Verwey transition (from cubic to monoclinic magnetite)[15], is shifted from ~120 K to values around ~105 K, indicating either partial oxidation[14] of magnetite or a minor fraction of $Ti^{4+}$ ions included in the magnetite crystal lattice[16]. Samples, 489.6 cm (NbrOCp), 613.7 cm (NbrOCp), 629.6 cm (NbrOCp), 645.7 cm (NdgOCr) and 696.3 cm (NdgOCrSid) show an evenly but varying decrease in remanence. For clarification, the gradients of the (Z)FC curves from Supplementary Figure 6b are shown in Supplementary Figure 6c.

The results of the **FC** measurements (Supplementary Fig. 6a and 6b) of the 445.4 cm (NbrOCp) and 454.7 cm (RbrOCp) samples are very similar to each other showing an evenly decrease with temperature. Relatively high remanence values remain at 300 K. Samples 461.6 cm (NlgOCp) and 479.7 cm (RbrOCp), are also relatively similar to each other. In the range between 50 and 150 K, their remanences do not decrease as much as those of the two previous samples, while sample 489.6 cm (NbrOCp) shows a temperature behavior that is within the variations of all previous four samples. However, its remanence value at 300 K is significantly lower. Samples 613.7 cm (NbrOCp) and 629.6 cm (NbrOCp) behave similarly at temperatures above ~170 K. They have then lost most of their remanence. In sample 613.7 cm (NbrOCp), the remanence decreases significantly up to about 100 K (more than in samples 445.4 cm (NbrOCp) and 454.7 cm (RbrOCp)), while in sample 629.6 cm (NbrOCp) the remanence decreases more slowly (also when compared to samples 445.4 cm (NbrOCp) and 454. 7 cm (RbrOCp)), continuing up to 150 K. Samples 645.7 cm (NdgOCr) and 696.3 cm (NdgOCrSid) show the most prominent decrease in remanence at low temperatures up to about 40 K with a sharp bend for sample 696.3 cm (NdgOCrSid) that can be probably explained by the presence of siderite[17]. At higher temperatures the remanences of both samples decrease similarly and almost uniformly. At 300 K, samples 445.4 cm (NbrOCp), 454.7 cm (RbrOCp), 461.6 cm (NlgOCp), and 479.7 cm (RbrOCp) retain significantly higher fractions of their initial remanences (about 10%), while those of the other samples decreases to ≤5%.

After RT-SIRM cycling (Supplementary Fig. 6a and 6d), samples 445.4 cm (NbrOCp), 454.7 cm (RbrOCp), 461.6 cm (NlgOCp), 479.7 cm (RbrOCp), and 489.6 cm (NbrOCp) show a similar loss of remanence of about 20% at 300 K. The cooling and rewarming curves meet at temperatures sometimes well below ~120 K, that characterizes the Verwey transition of magnetite. This indicates partial, but still low oxidation of the samples. The temperature behavior of sample 489.6 cm (NbrOCp) looks similar to that of 497.7 cm (RbrOCp), but shows no Verwey transition. Its RT-SIRM increases slightly more at lower temperatures, which could be explained by magnetic ordering of very small superparamagnetic particles at very low temperatures (<10 K). All curves seem to show a mixture of the behavior of magnetite and partially oxidized magnetite. This could be explained by surface oxidation of the magnetite particles. The RT-SIRM memory is between 0.71 and 0.83, which is significantly lower than for partially oxidized magnetite (0.90 - 0.95), but higher than for stoichiometric magnetite (0.55) (ref. 14). This again suggests a mixture of magnetite and partially oxidized magnetite. Maghemite, i.e., the fully oxidized magnetite, appears to be absent.

Samples 613.7 cm (NbrOCp), 629.6 cm (NbrOCp), and 645.7 cm (NdgOCr) show a similar behavior to each other during RT-SIRM cycling (Supplementary Fig. 6a). Their RT-SIRM memories range between 0.81 and 0.91, and the cooling and rewarming curves meet at temperatures of about 100 K or below (613.7 cm (NbrOCp) and 696.3 cm (NdgOCrSid)) probably indicating a higher degree of oxidation. Their respective remanences are significantly increased at very low temperatures suggesting a higher proportion of very fine-grained, superparamagnetic particles. Sample 696.3 cm (NdgOCrSid), taken from the core's siderite maximum, shows a RT-SIRM cycling behavior similar to that of the samples from the 445-490 cm core depth range. It also has an RT-SIRM memory of 0.71, probably indicating relatively low oxidation.

Bulk geochemical properties (represented by OC content and color) seems to have only little influence on the magnetic inventory: samples samples 613.7 cm (NbrOCp), 629.6 cm (NbrOCp), and 645.7 cm (NdgOCr) show only gradual differences. For example, the results for samples 629.6 cm (NbrOCp) and 645.7 cm (NdgOCr) look quite similar to each other, although their FC warming curves are different. Both samples seem to have an increased proportion of superparamagnetic particles as suggested by the steep remanence decrease at very low temperatures. Sample 696.3 cm (NdgOCrSid ) exhibits features of both samples 479.7 cm (RbrOCp) and 489.6 cm (NbrOCp) in its RT-SIRM cycling behavior, complemented by the signal caused by the probable presence of siderite (particularly visible in the sharp bend at about 40 K in the FC curve).

When looking at the RPI and inclination records, it is obvious that in several instances RPI maxima coincide with the dark gray intervals (representing OC-rich, probably anoxic conditions; see discussion in the main text), and that the paleomagnetic reversals coincide mostly with beige-brownish (oxic) sediments (Fig. 2a). We found possible indications of coherence between the RPI datasets and their respective normalizers (ARM, IRM, magnetic susceptibility). However, among $RPI_{arm}$, $RPI_{irm}$ and $RPI_{sus}$, only one frequency in the case of $RPI_{irm}$ has a confidence interval completely above the 0.05 significance level (Supplementary Fig. 7). For all other frequencies, as well as for $RPI_{arm}$ and $RPI_{sus}$, the confidence intervals are too large for a reliable conclusion. However, we cannot completely rule out some bias that could modulate the amplitudes of the peaks and troughs in the RPI records.

In conclusion, a systematic influence of the changing magnetic properties on the magnetostratigraphy for core PS115-2-2, in the form of systematically different properties of normal and reversed samples, is not apparent. Differences between samples of either normal or reversed magnetic polarity are not greater than the differences between samples of the same magnetic polarity. Furthermore, the different OC content seems to have little, if any, effect on the magnetic properties of the sediment relevant for the magnetostratigraphic interpretation. OC-rich samples differ, if at all, only marginally in their magnetic properties from OC-poor samples. Thus, our inclination record can be considered as reliable record of the Earth's magnetic field, i.e., reflecting intervals of normal and reverse polarities.

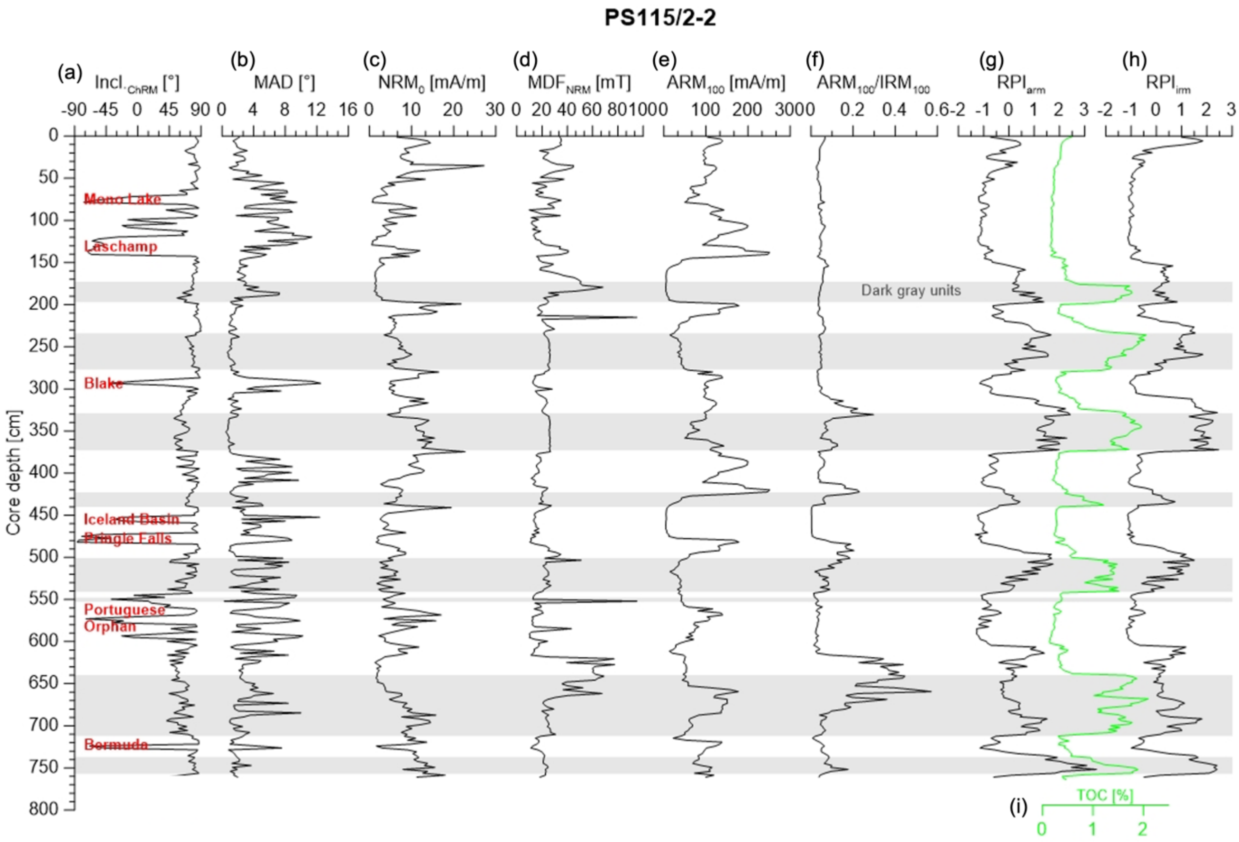


**Supplementary Fig. 5**

**Paleo- and rock magnetic parameters for Core PS115/2-2** (this study). (a) inclination of characteristic remanent magnetization with assigned geomagnetic excursions (after ref. 18); (b) maximum angular deviation (MAD); (c) intensity of natural remanent magnetization (NRM) at the 0 mT demagnetization level; (d) median destructive field of the NRM; (e) intensity of anhysteretic remanent magnetization (ARM) imparted in a 100 mT alternating magnetic field in presence of a 50 µT DC field; (f) ratio of anhysteretic remanent magnetization to isothermal remanent magnetization (IRM); (g) relative paleointensity (RPI) of the Earth's magnetic field based on ARM normalization; (h) relative paleointensity of the Earth's magnetic field based on IRM normalization, each in standardized form; and (i) content of total organic carbon (TOC). Gray color bars represent units of dark gray sediment color. Source data of this figure are provided as a Source Data file.

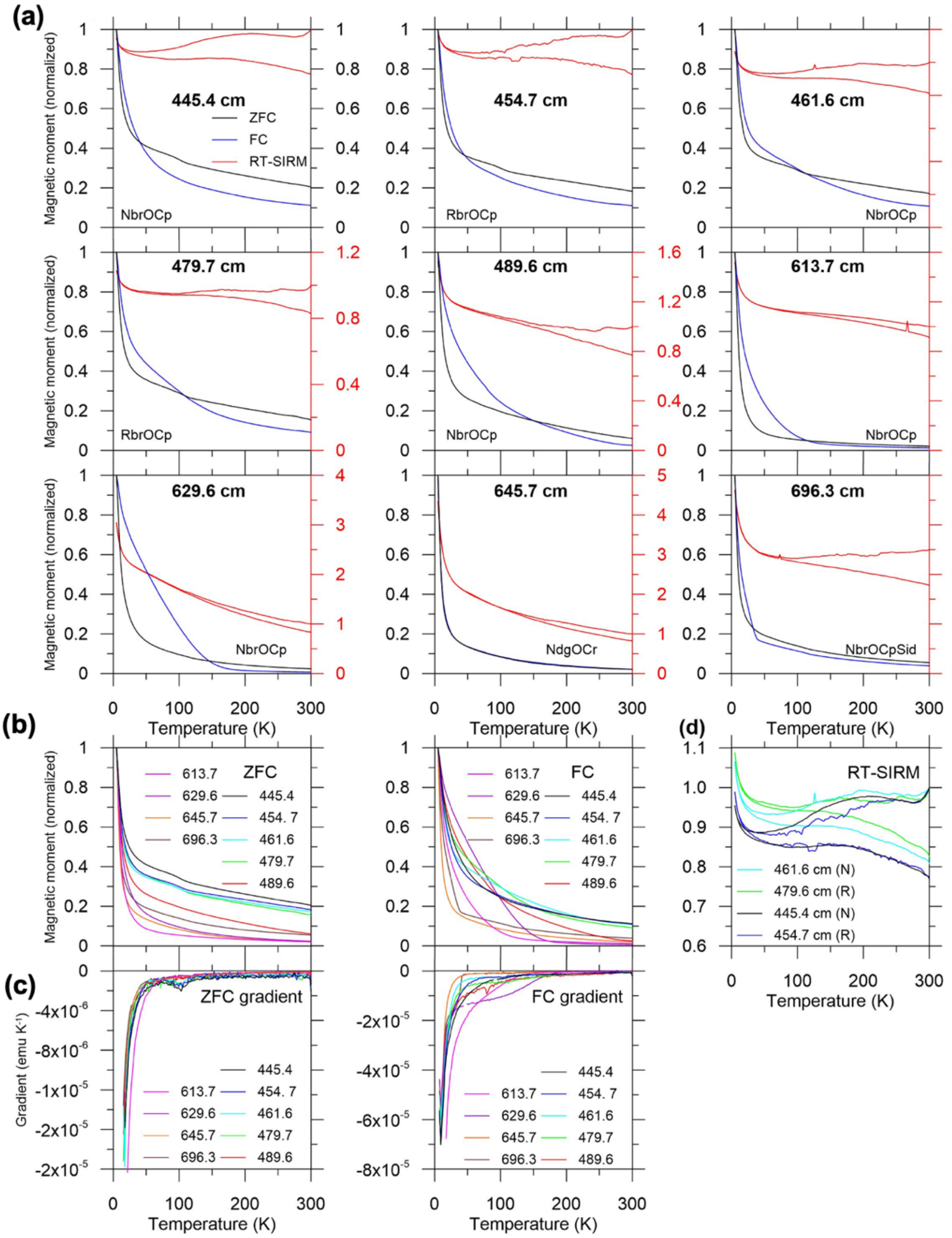


**Supplementary Fig. 6**

**Results of low-temperature magnetic remanence measurements** for nine selected samples from core PS115/2-2 of different magnetic polarities and from different geochemical environments: (a) zero-field warming curves of a remanence imparted at 5 K in a 5 T DC field after zero-field cooling (ZFC) from 5 K to 300 K, zero-field warming curves of a remanence imparted at 5 K in a 5 T DC field after cooling in a 5 T magnetic field (FC) from 5 K to 300 K, cooling and rewarming curves of a remanent magnetization acquired in a 5 T DC field at 300 K between 300 K and 5 K and back to 300 K (RT-SIRM cycling). (Z)FC curves are normalized to their respective initial value at 5K, RT-SIRM cycling curves to their respective initial value at 300 K. Codes: N, R - normal (reverse) magnetic polarity; br – brownish; lg, dg - light (dark) gray; OCp, OCr - total organic carbon poor (rich); Sid - siderite. (b) Normalized (Z)FC curves for all samples. (c) Gradients of respective (Z)FC curves. (d) Normalized cooling and rewarming curves of a remanent magnetization acquired in a 5 T DC field at 300 K between 300 K and 5 K and back to 300 K (RT-SIRM cycling) for two samples of normal and two samples of reverse magnetic polarity.

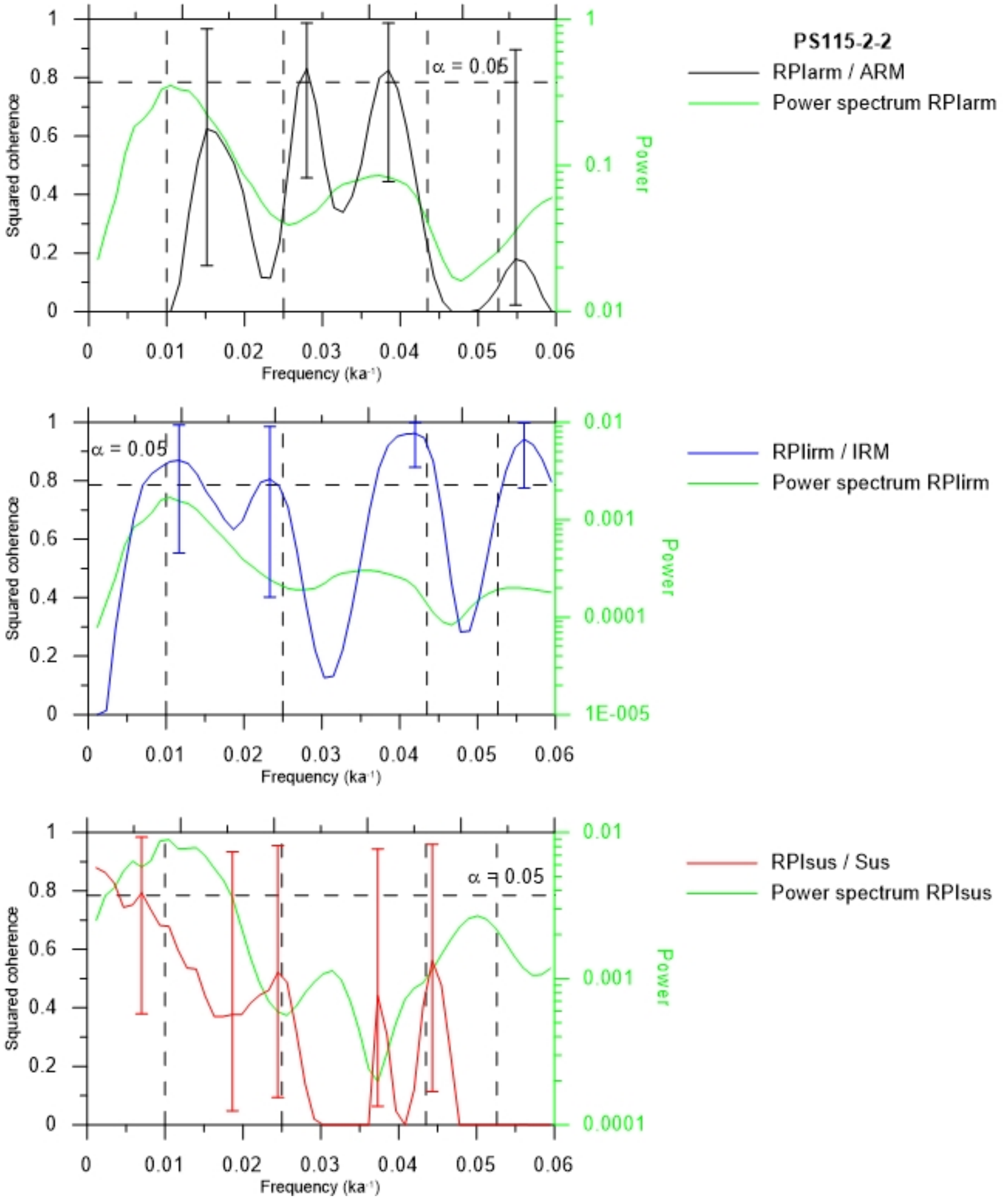


**Supplementary Fig. 7**

**Results of computation of coherency with the software Spectrum**[19] for RPI records ($RPI_{arm}$, $RPI_{irm}$, $RPI_{sus}$) with their respective normalizers anhysteretic remanent magnetization (ARM), isothermal remanent magnetization (IRM), and magnetic susceptibility (SUS). Vertical dashed lines denote cycles at 100, 40, 23 and 19 kyr periods.

### (3) Supplementary information of correlation of RPI records with tuning target and alternative age model (Supplementary Figure 8)

As outlined in the main text, our relative paleo-intensity (RPI) record is tuned to the global RPI/isotope stack “PISO-1500” that is based on coupled RPI and oxygen isotope records and developed as stratigraphic template for correlating and dating sedimentary records for the last 1.5 Myr (ref. 20; for the last 450 kyr of the stack see Fig. 2b and Supplementary Fig. 8). Both RPI records show a quite similar pattern, and an alignment of the records is possible, supporting our proposed age model. However, there is one ambiguity. In the PS115/2-2 RPI record, a very prominent RPI peak occurs at a depth of about 425-440 cm that looks very similar to a peak around 205 ka in the PISO-1500 stack (Peak “7a” in Fig. 2b). This correlation mismatches with the interpretation of the inclination record in which we assign the interval of 452-457 cm core depth to the Iceland Basin geomagnetic excursion and therefore an age of 188 ka (Fig. 2a). This means that both timestamps (RPI and inclination) did not occur contemporaneously. This discrepancy can be explained by a minor hiatus that may occur at a core depth of about 373 cm where RPI values abruptly change from minimum to maximum values (Fig. 2a). If present, this hiatus would represent the time interval between about 190 and 170 ka (Supplementary Fig. 8). Such a hiatus might be supported by the RPI record (Tie point “7a” in Fig. 2b) and $^{230}Th_{ex}$ maxima correlated with MIS 7, and would suggest that the Iceland Basin geomagnetic excursion is missing (alternative age model; Supplementary Fig. 8d and 8e). If this alternative age model is correct, however, this would not affect our main data interpretation and paleoenvironmental reconstructions.

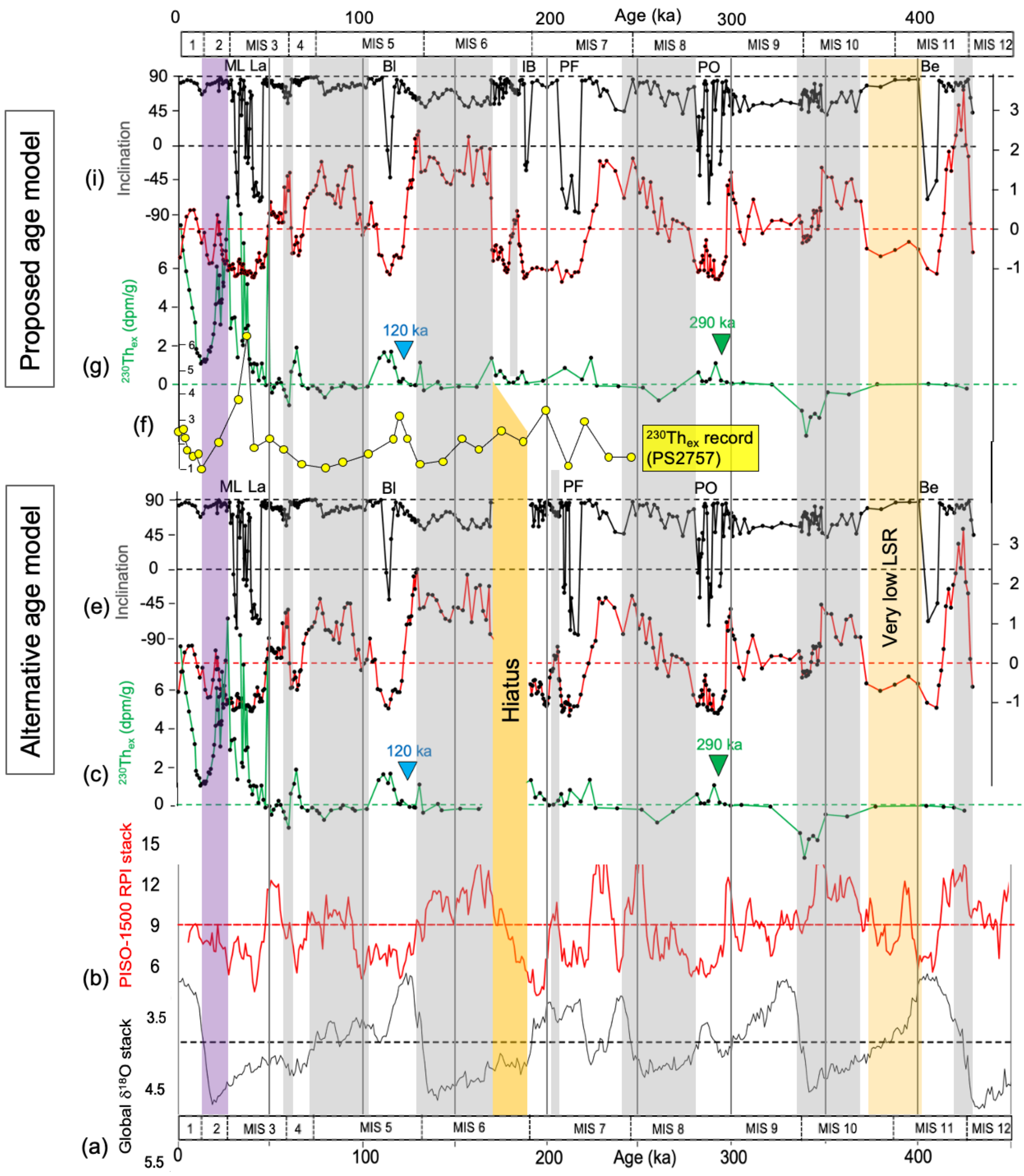


**Supplementary Fig. 8**

**Proposed age model (used in this study) and alternative age model of Core PS115/2-2**. (a) Global benthic oxygen isotope stack[21]. The OC-1 to OC-7 intervals are highlighted as gray bars, the Last Glacial Maximum (LGM) characterized by OC-poor sediments is highlighted by a purple bar. (b) Global virtual axial dipole moment (VADM)-calibrated PISO-1500 relative paleointensity (RPI) stack[20]. (c-e) $^{230}Th_{ex}$, relative paleointensity (RPI), and paleomagnetic inclination records for Core PS115/2-2 plotted versus the alternative age model (this study). (f) Yellow circles show a low-resolution $^{230}Th_{ex}$ record from Core PS2757 (ref. 11; for core location and lithology see Supplementary Fig. 4) that correlate quite well with our higher-resolution record. (g-i) $^{230}Th_{ex}$, relative paleointensity (RPI), and paleomagnetic inclination records for Core PS115/2-2 plotted versus our proposed age model. Labelled geomagnetic excursions after ref. 18; for full names see Figure 2a. Green and blue triangles indicate the $^{230}Th_{ex}$ and $^{231}Pa_{ex}$ extinction ages. For further details concerning the age models see text. Source data of Supplementary Figure 8c-8d and 8g-8i are provided as a Source Data file.

## (4) Supplementary information on organic carbon source and maturity as well as sediment anoxia (Supplementary Figures 9 to 10)

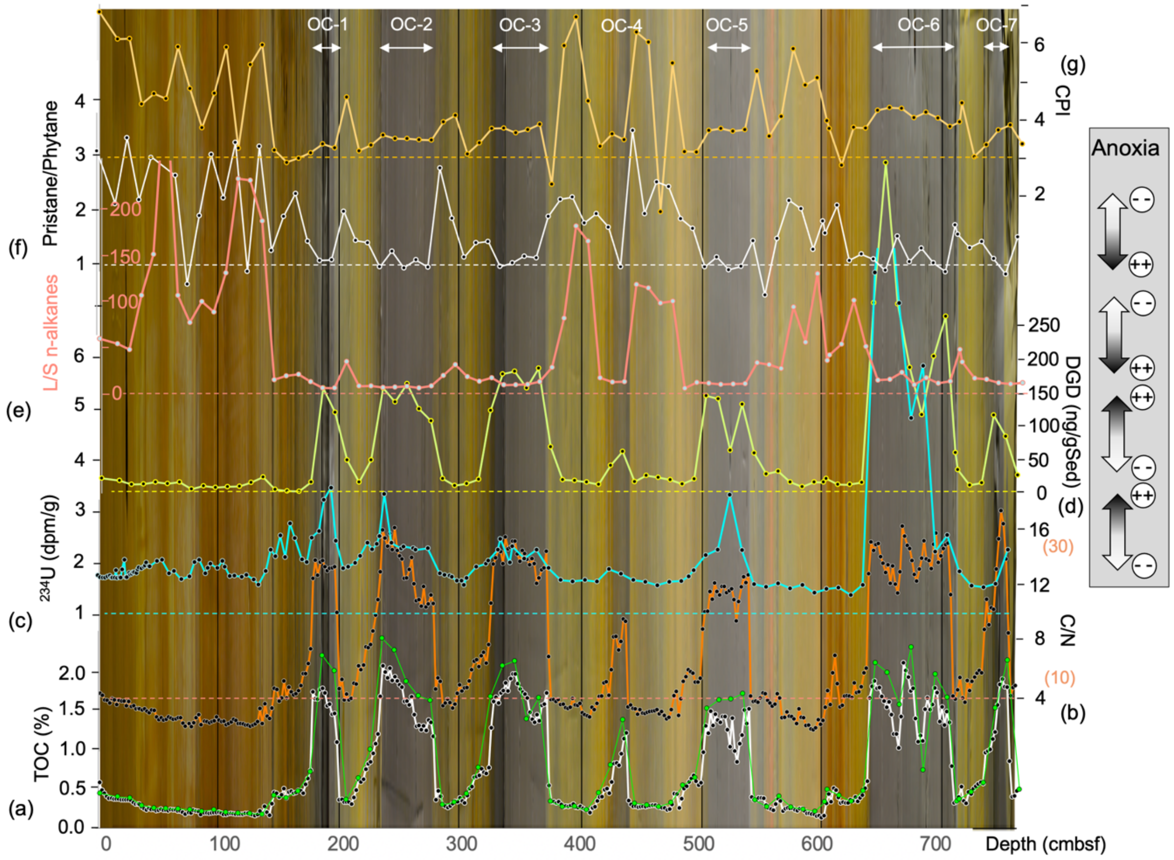


**Supplementary Fig. 9**

**Organic-geochemical proxy records at Core PS115/2-2, plotted vs depth** (all data this study)**.** (a) Total organic carbon (TOC) content; (b) C/N ratios calculated as TOC/TN ratios and used as a first-order proxy for estimating the marine (C/N = 5 to 8) and terrestrial (C/N >>10) proportions of the OC - when corrected for inorganic nitrogen the maxima of 14 to 16 would even increase to >25 (orange number in brackets; see Methods); (c) $^{234}U$ concentrations indicative for anoxic conditions; (d) $C_{33}$ dialkyl glycerol diether (DGD) as proxy for anaerobic bacteria[22]; (e) ratio of long-chain and short-chain (L/S) *n*-alkanes indicative for the ratio of terrestrial higher plants vs. algae-type OC, (f) pristane/phytane ratio indicative for sediment anoxia[23,24] and (g) carbon preference index (CPI) as indicator for OC source and maturity[25-27]. As background of the records, the emerged photograph of the PS115/2-2 sedimentary sequence (see Fig. 3) is shown, highlighting the dark gray OC-rich intervals OC-1 to OC-7. See text for further details. Source data of this supplementary figure are provided in the source data files of Figures 4g, 4h, 6c and 6g).

In the TOC record of (a) based on 381 data points, a lower-resolution record based on 79 data points, is added in green. This record produced as part of a master thesis in 2018/2019 (ref.5), indicates that the general features of the high-resolution record are already represented in the lower-resolution record. Furthermore, it seems to be obvious that the values from OC-poor intervals are more or less identical in both records whereas the values from the OC-rich intervals are higher in the green curve but by 0.2 to 0.4% lower in the white high-resolution curve produced in 2021/2022. Here, one could speculate that parts of the more labile (fresh) organic compounds in the OC-rich intervals might have been already decomposed during the three additional years of storage, causing lower TOC contents.

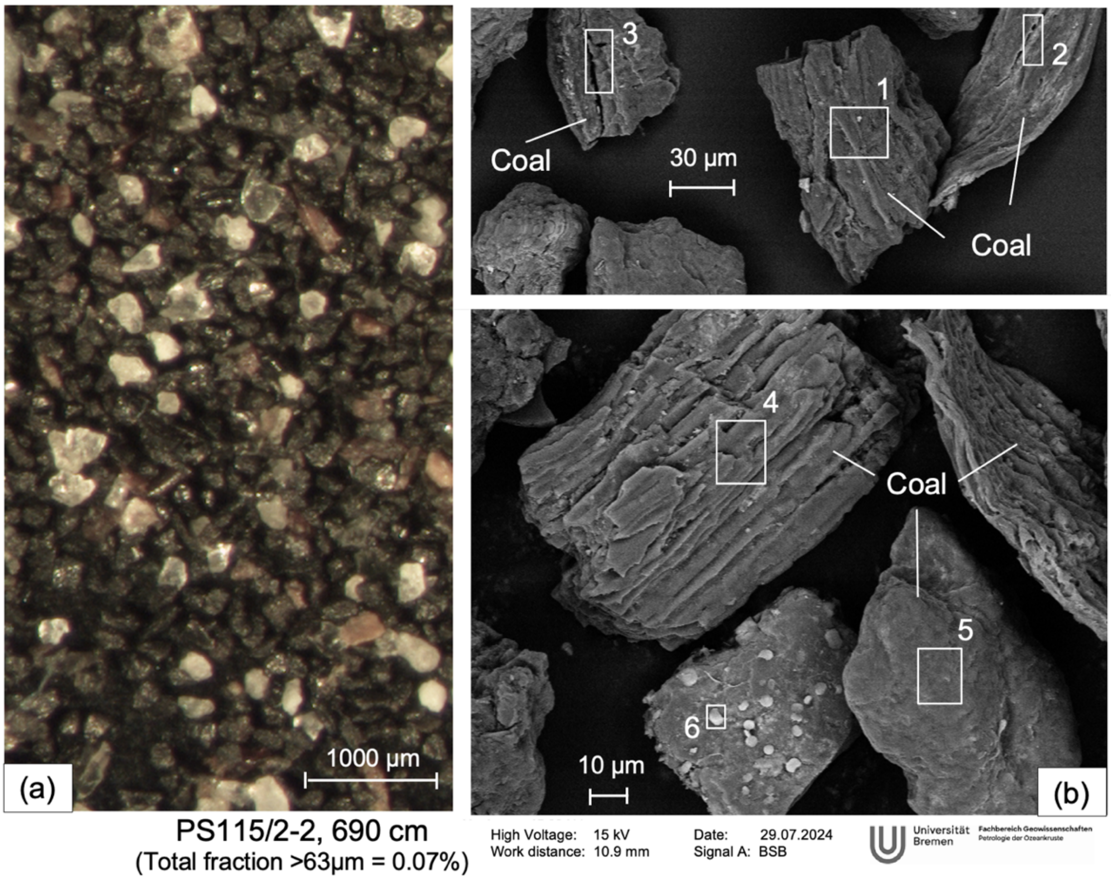


(c) EDAX

| PS115/2-2-690 | Spectrum 1 | Spectrum 2 | Spectrum 3 | Spectrum 4 | Spectrum 5 | Spectrum 6 |
|---|---|---|---|---|---|---|
| | Atom (%) | Atom (%) | Atom (%) | Atom (%) | Atom (%) | Atom (%) |
| Carbon | 70,32 | 49,32 | 62,08 | 65,51 | 68,42 | 22,46 |
| Oxygen | 26,26 | 39,74 | 31,56 | 29,72 | 29,01 | 60,20 |
| Sodium | - | 0,29 | - | - | - | 0,73 |
| Magnesium | 0,33 | 0,65 | 0,43 | 0,32 | 0,24 | 0,67 |
| Aluminum | 0,54 | 2,50 | 0,89 | 0,25 | 0,23 | |
| Silica | 0,76 | 5,07 | 1,54 | 0,38 | 0,32 | 0,62 |
| Sulphur | 0,20 | 0,15 | 1,01 | 1,07 | 0,41 | 6,34 |
| Chlorine | 0,10 | 0,07 | - | - | - | 0,60 |
| Potassium | - | 0,47 | - | - | 1,26 | 0,13 |
| Calcium | 1,50 | 0,94 | 2,15 | 2,23 | - | 8,25 |
| Titanium | - | - | - | 0,21 | - | - |
| Iron | - | 0,79 | 0,34 | 0,31 | 0,12 | - |
| | 100 | 100 | 100 | 100 | 100 | 100 |
| | | | | | | |
| | Coal | Coal | Coal | Coal | Coal | Gipsum/Anhydrite |
| | | | | | | (surrounding: coal) |

**Supplementary Fig. 10**

**SEM photograph and EDX data of coal particles from the OC-6 interval** (PS115/2-2-690). (a) Overview photograph of coarse fraction >63 m of sample PS115/2-2-690 cm, showing the predominance of fine-sand-sized dark/black grains. Note: the total amount of sand is only 0.07% of the total sediment, i.e., the bulk sediment is a silty clay. (b) Scanning electron microscope (SEM) photograph of selected dark/black particles. The six areas analyzed by EDX are marked by white squares. (c) Table summarizing the elemental composition derived from EDX analysis. Based on the element spectra 1 to 5, the particles have been identified as coals. Spectrum 6 indicates a gipsum/anhydrite crystal/particle (surrounded by coal). The SEM and EDX analyses were carried out in the laboratory of the Research Group "Petrology of the Ocean Crust" (Faculty of Geosciences, Bremen university) that is equipped with the field emission electron microscope SUPRA 40 from the company Zeiss and a Bruker XFlash 6|30 EDX detector.

## (5) Supplementary information on mineralogical proxy records, source regions and glacial deposits (Supplementary Figures 11 to 17)

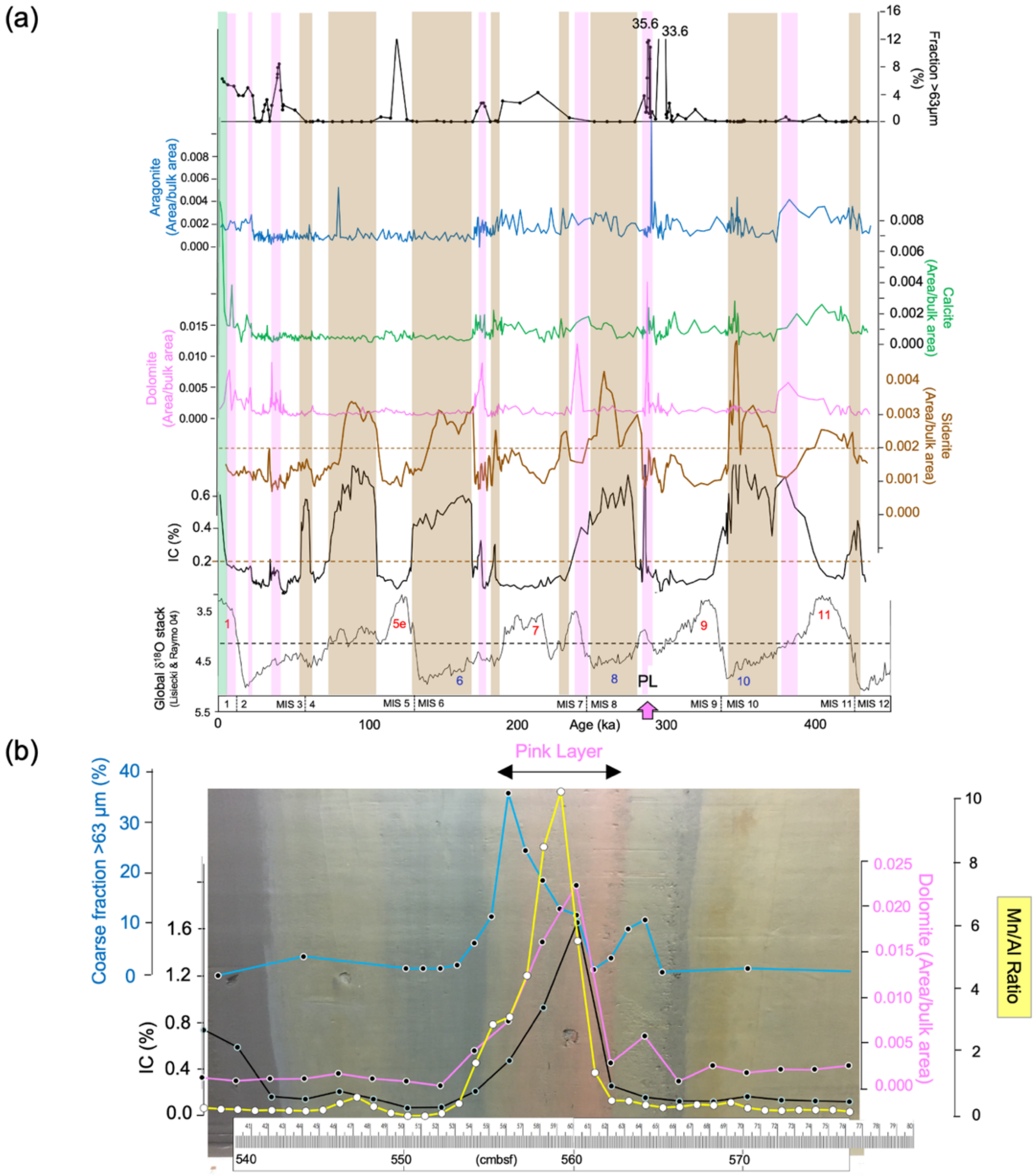

Supplementary Fig. 11

**Inorganic carbon (IC) content, relative concentrations of main carbonate minerals, and percentages of coarse fraction >63 µm** of Core PS115/2-2 (This study). (a) Global benthic oxygen isotope stack[21] and IC content and relative siderite, dolomite, calcite, and aragonite contents, respectively, expressed as ratio of the XRD single mineral peak intensity vs. sum of total analyzed intensities (see Methods for details), and coarse fraction >63 µm, all plotted vs. age. The absolute dolomite maximum that correlates with maximum content of coarse fraction, represents the pink layer (PL), probably correlating with the pink-white layer PW2 (see Supplementary Figure 17). Based on our age model, this layer is dated to about 282 ka (i.e., late MIS 9/early MIS 8). Intervals characterized by elevated contents of siderite, dolomite and calcite are highlighted by transparent brownish, pink, and green background colours, respectively. The siderite maxima coincide with the dark gray OC-rich intervals (cf., Figs. 4 and 6). (b) Contents of IC, dolomite and coarse fraction as well as the Mn/Al ratio from XRF scanning of the interval from 538 to 576 cm core depth, representing the core section around the prominent pink layer. As background, a photograph of the core lithology is shown. Source data of this figure are provided at https://doi.org/10.1594/PANGAEA.975790 .

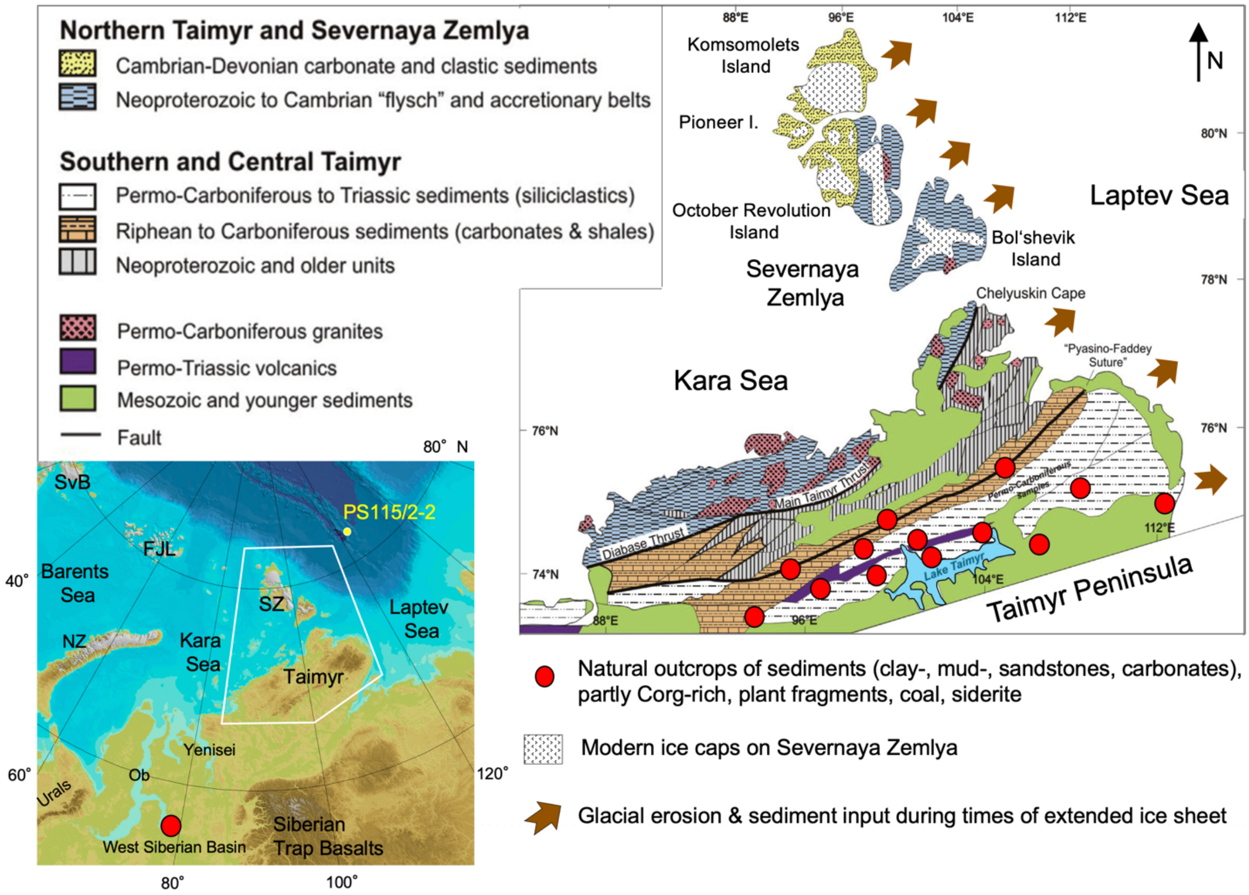


**Supplementary Fig. 12**

**Simplified geological map of Taimyr Peninsula and Severnaya Zemlya** (from ref. 28, supplemented). Most probable source areas of glacial erosion and sediment input are Paleozoic and Mesozoic sedimentary rocks (clay-, mud and sandstones, carbonates)[28-30]. Red circles indicate locations of natural outcrops of sequences of sedimentary rocks (i.e., clay-, mud-, sandstones, carbonates) that are partly characterized by elevated OC contents, plant fragments, coal, and siderite[31,32]. The bathymetric map is extracted from the IBCAO International Bathymetric Chart of the Arctic Ocean (IBCAO)[3].

NZ Novaya Zemlya, SZ Severnaya Zemlya, FJL Franz Josef Land, SvB Svalbard.

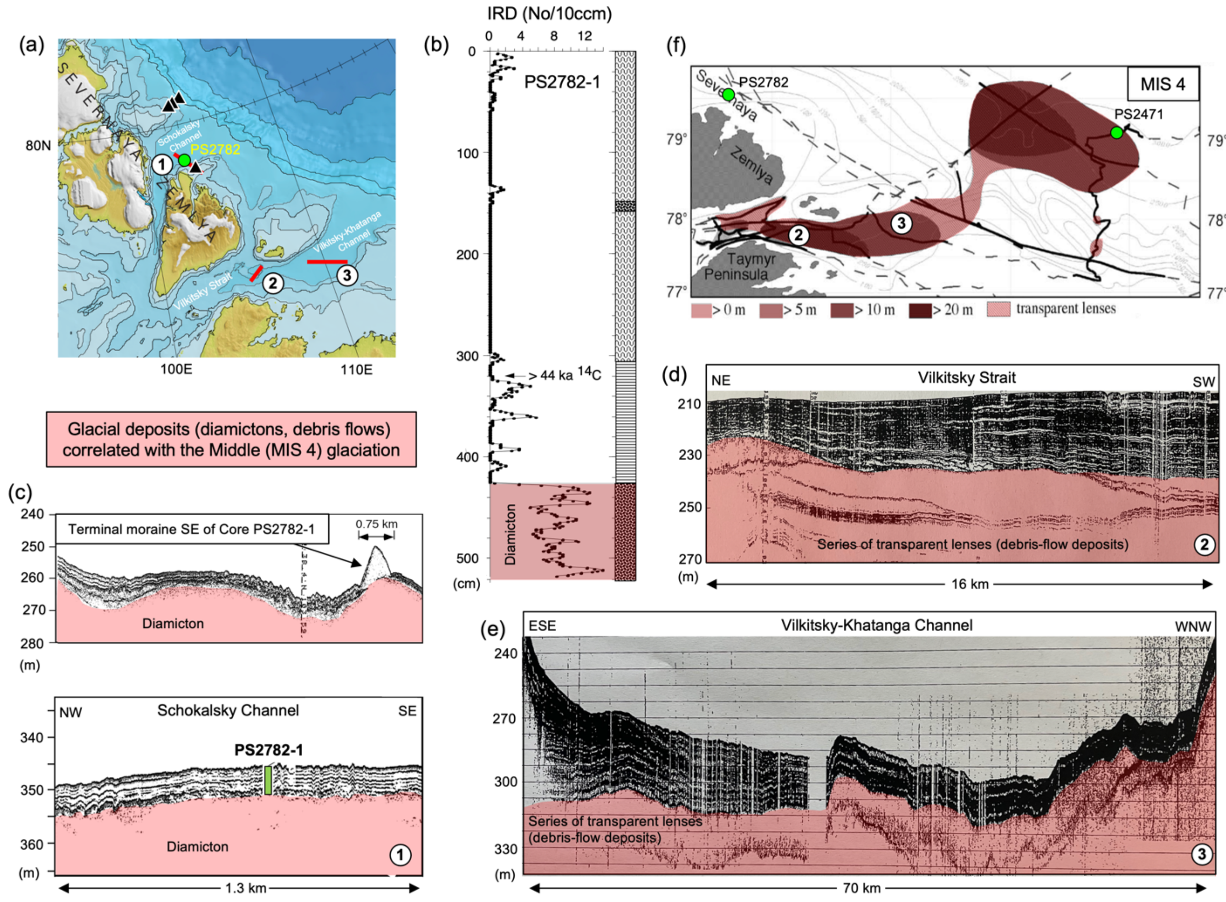


**Supplementary Fig. 13**

**Glacial deposits mapped by Parasound survey and correlated with the MIS 4/3 glaciation**. (a) Bathymetric map (extracted from the IBCAO map[3]) of the area around Severnaya Zemlya with locations of Core PS2782, mapped end moraines (black tringles), and Parasound profiles 1 (Schokalsky Channel), 2 (Vilkitsky Strait) and 3 (Vilkitsky-Khatanga-Channel). (b) Lithology and numbers of IRD grains >2 mm per 10 cc in Core PS2782-1 obtained off Severnaya Zemlya (340 m of water depth)[33]. An AMS$^{14}$C dating of >44 ka indicates that the diamicton is of pre-late Weichselian (MIS 4?) age. (c-e) PARASOUND profiles (c) across the Core PS2782 location and across a terminal moraine structure found very close to the core location, (d) the Vilkitsky Strait, and (e) the Vilkitsky-Khatanga-Channel, indicating a series of transparent lenses, glacigenic processes during MIS 4 (refs. 33-35; Niessen, unpubl. data 1997). (f) Bathymetric map showing the distribution and the related thicknesses of the transparent facies of seismic unit IV correlated with ice-sheet advance in MIS 4 (ref. 35). Grey, stippled lines show the cruise track of the RV Polarstern[34]. The MIS 4 glacial deposits are highlighted by pink background color.

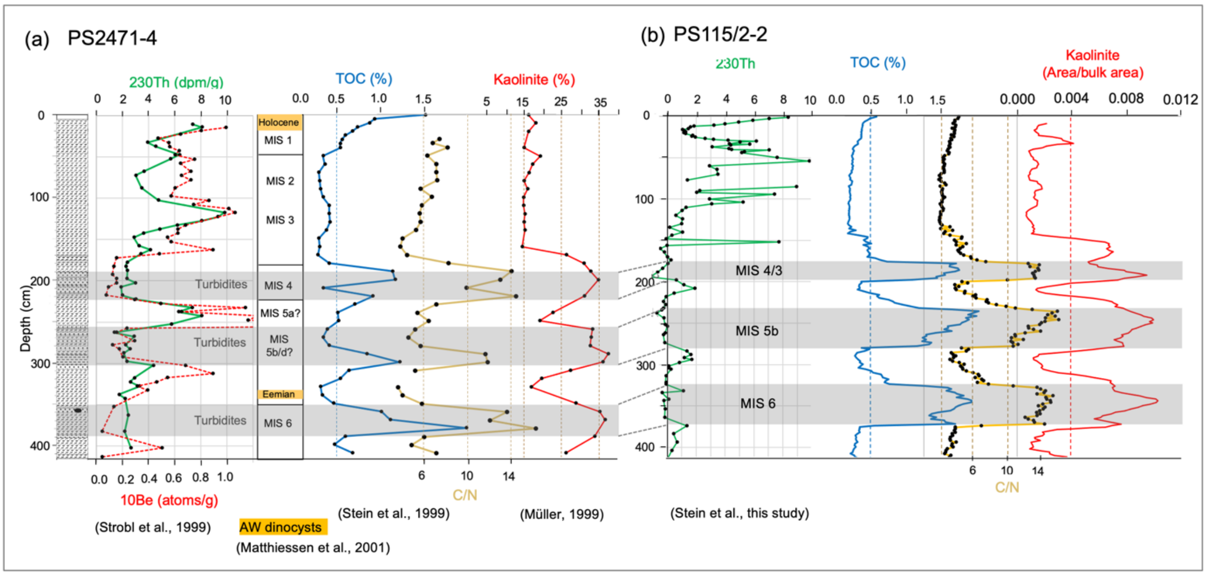


**Supplementary Fig. 14**

**Sediment proxy records (MIS 6 to MIS 1): Core PS2471-4 vs. Core PS115/2-2**. For core location see Figure 1. (a) Core PS2471-4 (representing more proximal conditions): $^{230}$Th and $^{10}$Be records[36], total organic carbon (TOC) content and C/N ratios[37], and kaolinite content of the clay fraction <2µm[38]. Occurrence of Atlantic Water (AW) dinocysts indicative for the Eemian and Holocene intervals, respectively, are highlighted by orange background color[39]. (b) Core PS115/2-2 representing more distal conditions): $^{230}$Th, TOC content, C/N ratios, and relative kaolinite content expressed as ratio of the XRD kaolinite peak intensity vs. sum of total analyzed intensities of the bulk sediment (This study; for data see source data files of Figures 4g, 4h, 6c and 6g). The kaolinite record is presented as three-point-moving average.

In both cores, maxima in TOC, C/N and kaolinite representing mass-wasting processes and sediment input from Eurasia during glacial (stadial) intervals, can be correlated perfectly.

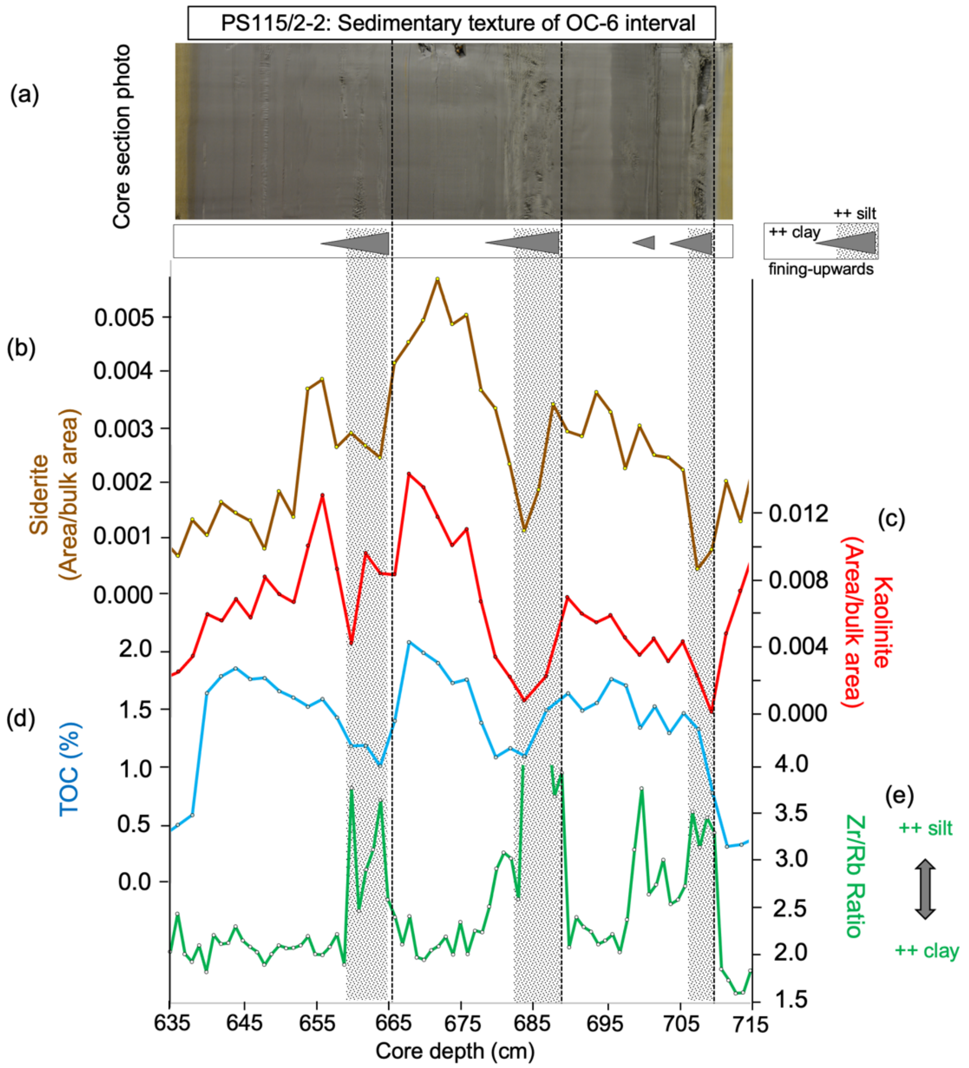


**Supplementary Fig. 15**

**Proxy records for sediment provenance and transport processes** of the PS115/2-2 depth interval 635-715 cm, representing the dark gray OC-6 interval (This study). (a) Core photograph showing internal sedimentary structures with fining upward from silt to clay[4]; (b and c) relative concentrations of siderite and kaolinite; (d) TOC content; (e) Zr/Rb ratios as indicator for grain size (transport energy). Source data of Supplementary Figure 15b-15e are provided as a Source Data file.

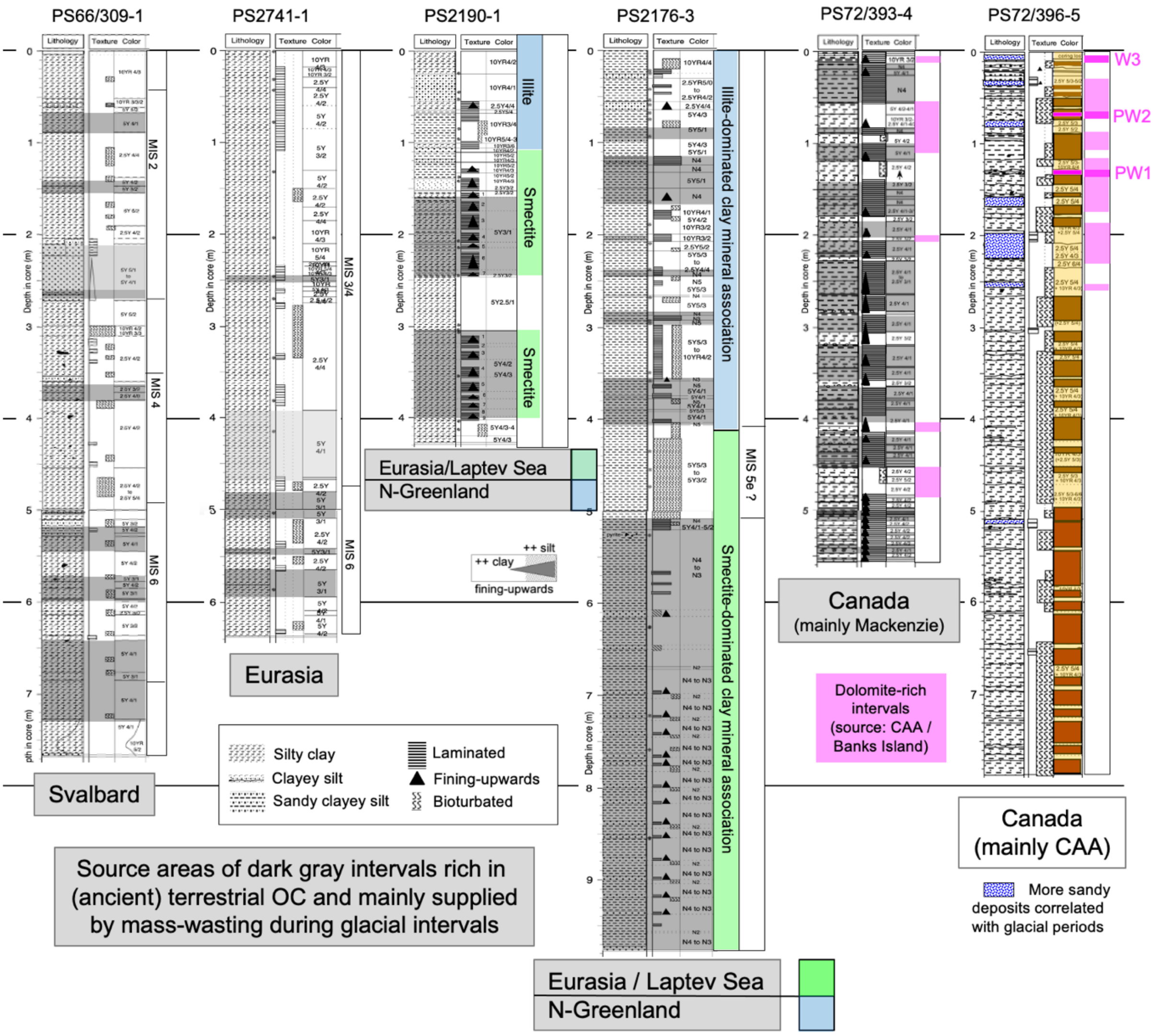


**Supplementary Fig. 16**

**Lithostratigraphy of selected Arctic Ocean deep-sea sediment cores** characterized by prominent dark gray (OC-rich) intervals (highlighted by gray background color), glacigenic processes and detrital sediment input during glacial intervals. Potential source areas of the detrital sediment fraction are indicated. Information on lithostratigraphy and partly still tentative age models is extracted from the cruise reports: Core PS66/309-1 (ref. 40); PS2741-1 (ref. 6); PS2190-1 and PS2176-3 (ref. 41), and PS72/393-4 (ref. 42). For PS2190-1 and PS2176-3, a change in source areas from Eurasia/Laptev Sea continental margin to Greenland is proposed based on changes in the clay-mineral assemblages[43]. Data on organic carbon contents of these cores are published: Core PS66/309-1 (ref. 44), Core PS2741-1 (ref. 45), Cores PS2190-1 and PS2176-3 (refs. 46, 47). In contrast, the lithostratigraphic record of Mendeleev Ridge Core PS72/396-5 is shown, characterized by glacial/interglacial changes in lithology and prominent horizons with maximum dolomite content (i.e., pink-white layers PW1 and PW2 and white layer W3), but OC-poor sediments throughout[48]; for further details see Supplementary Figure 17. These differences display that the geology of the ice-covered hinterland is a primary factor influencing whether glacial OC-rich or OC-poor sediments are transported towards the deep-sea basins. For further details and references see text.

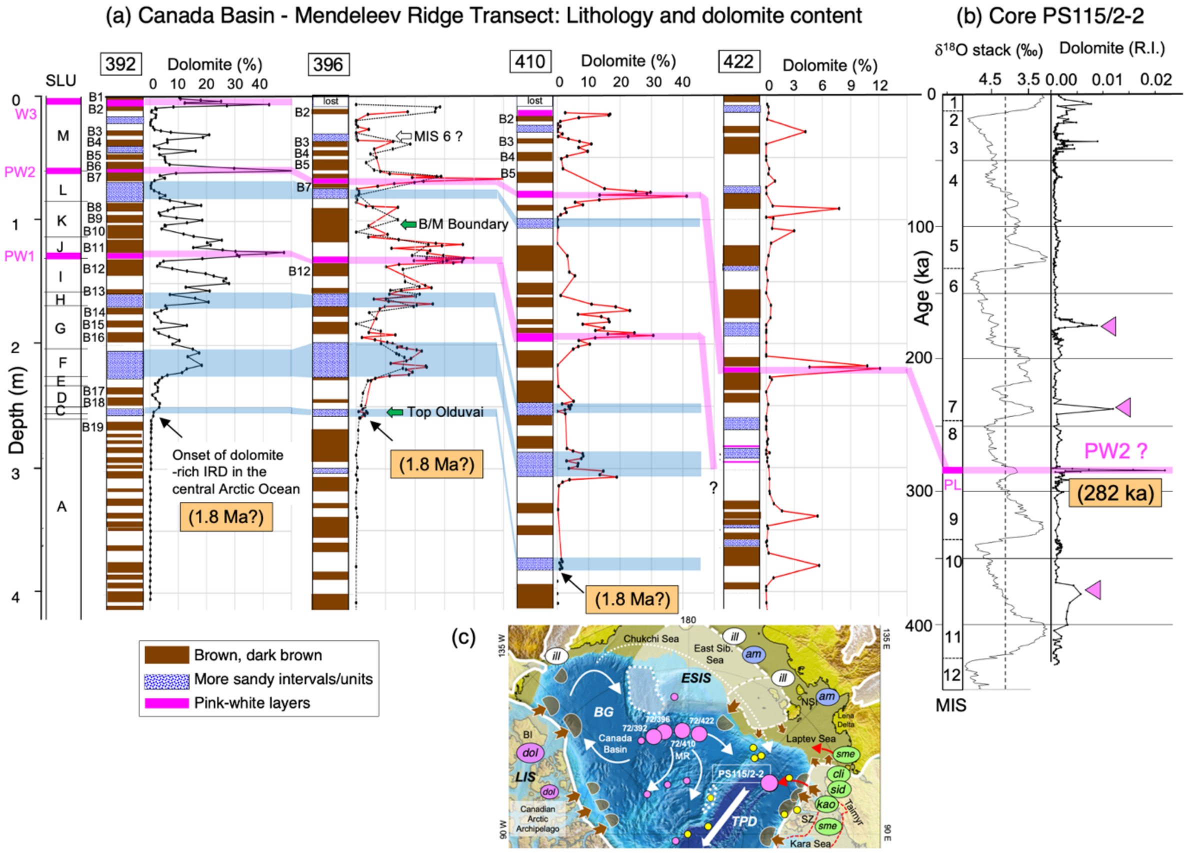


**Supplementary Fig. 17**

**Simplified scheme of the occurrence of brown, dark brown, and dark reddish brown layers, more sandy intervals/units, and main pink-white layers and dolomite** content in selected sediment cores from a transect across Mendeleev Ridge; for core locations see map (c). Standard lithostratigraphic units (SLU) A to M (according to ref. 49) and depths of main pink-white layers PW1, PW2 and W3 and brown layers B1-B19 are indicated[48,50]. The more sandy units L, J, H, F, and C are correlated with major glaciations[48-50]. The first input of coarse-grained, dolomite-rich IRD (Unit C) is interpreted as onset of major glaciations in northern Canada with ice sheets reaching the Arctic shelf break. Dolomite records: 392/black[50], 396/ black stippled[51], and 396, 410 and 422/red[52-55]; for XRD analytics and data evaluation see refs 50 and 52). Based on correlation with well-dated detrital carbonate (dolomite) records from the North Atlantic[56,57] and the assumption that the extended Laurentide Ice Sheet (LIS) reached contemporaneously the North Atlantic and Arctic shelf breaks, Stein et al. (ref. 48) have proposed that this event occurred during the prominent MIS 16 glaciation. Based on new paleomagnetostratigraphic data (green arrows: Top Olduvai and Brunhes/Matuyama (B/M) boundary[58,59]) supporting earlier work by Jones (ref. 60), and $^{230}$Th data (open arrow: MIS 6; ref. 61) from Core PS72/396, however, an age of about 1.8 Ma seems to be more probable. That means, the onset of major glaciations with extended North American ice sheets and detrital sediment input into the Arctic Ocean occurred much earlier than previously thought. (b) The prominent pink layer determined in Core PS72/115/2-2 and characterized by maximum content of coarse-grained dolomite (see Supplementary Fig. 11) probably represents the pink-white layer PW2 dated to 282 ka. Further minor dolomite peaks are highlighted as pink triangles. Global benthic oxygen isotope stack[21]. (c) Map showing core locations, extent of MIS 6 ice sheets, and mineralogical characteristics of circum-Arctic geology (extracted from Fig. 1; for further details and references see Fig. 1). Dolomite data of Supplementary Figure 17a and 17b are provided as a Source Data file.

## (6) **Supplementary Tables**

### Supplementary Table 1

**AMS$^{14}$C datings carried-out with the Accelerator Mass Spectrometry MICADAS system** at the Alfred Wegener Institute in Bremerhaven[62].

(a) Three samples of the planktic foraminifer (*Neogloboquadrina pachyderma* sinistral) taken from the box corer (GKG) PS115/2-2-1. Using the radiocarbon calibration program CALIB 8.20 and the Marine 20 calibration curve with a global mean reservoir age (R = 550 yrs.) (ref. 63), and a local marine reservoir correction (deltaR) value of 345 +/- 60 yrs (ref. 64), calendar ages were calculated.

(b) Due to the lack of biogenic carbonate (foraminifers), seventeen samples of bulk acid-insoluble OC (AIOC) residues (with sample weights >300 µgC) were selected from the box corer (GKG) core (3 to 27 cm depth) and Kastenlot (KAL) core (34 - 178 cm depth) for AMS$^{14}$C dating. As the exact composition of the AIOC (i.e., marine vs. terrestrial OC, fresh vs. reworked OC) is not known, reservoir corrections have to be taken with caution. Thus, uncalibrated $^{14}$C ages of the AIOC are shown in Figure 3. In this table, however, also calibrated ages are listed. These are based on the assumption (!) that the AOIC is predominantly terrestrial and, thus, no marine reservoir correction was done. Then, the radiocarbon calibration program CALIB 8.20 and the InterCal20 calibration curve[65] were used and calendar ages calculated.

**(a) Dating of planktic foraminifers (*N. pachyderma*)**

| AWI-MICADAS | Gear | Depth | uncalib. age | +/- error | ΔR | +/- error | Median Probability | 1 sigma lower | 1 sigma upper | 2 sigma lower | 2 sigma upper |
|---|---|---|---|---|---|---|---|---|---|---|---|
| No | | (cm) | (yrs.) | (yrs.) | (yrs.) | (yrs.) | (Cal. yrs. BP) | (Cal. yrs. BP) | (Cal. yrs. BP) | (Cal. yrs. BP) | (Cal. yrs. BP) |
| 8993.1.1 | GKG | 0,5 | 1109 | 71 | 345 | 60 | 221 | 90 | 326 | 0 | 414 |
| 6481.1.1 | GKG | 3 | 2093 | 62 | 345 | 60 | 1141 | 1046 | 1259 | 926 | 1335 |
| 9532.1.1 | GKG | 5 | 3864 | 75 | 345 | 60 | 3233 | 3100 | 3371 | 2942 | 3489 |

**Remarks: Radiocarbon calibration program CALIB 8.20; Marine20 calibration curve with global reservoir R = 550 yrs**

**(b) Dating of bulk organic carbon**

| AWI-MICADAS | Gear | Depth | uncalib. age | +/- error | ΔR | +/- error | Median Probability | 1 sigma lower | 1 sigma upper | 2 sigma lower | 2 sigma upper |
|---|---|---|---|---|---|---|---|---|---|---|---|
| No | | (cm) | (yrs.) | (yrs.) | (yrs.) | (yrs.) | (Cal. yrs. BP) | (Cal. yrs. BP) | (Cal. yrs. BP) | (Cal. yrs. BP) | (Cal. yrs. BP) |
| 9520.1.1 | GKG | 3 | 8881 | 54 | - | - | 10006 | 9982 | 10050 | 9763 | 10189 |
| 9779.1.1 | GKG | 11 | 15656 | 49 | - | - | 18914 | 18860 | 18956 | 18825 | 19034 |
| 9780.1.1 | GKG | 19 | 19070 | 60 | - | - | 22992 | 22944 | 23032 | 22898 | 23098 |
| 9521.1.1 | GKG | 27 | 19040 | 81 | - | - | 22975 | 22916 | 23034 | 22722 | 23150 |
| 9781.1.1 | KAL | 34 | 22290 | 80 | - | - | 26681 | 26661 | 26876 | 26359 | 26924 |
| 9782.1.1 | KAL | 42 | 28180 | 166 | - | - | 32202 | 31823 | 32275 | 31740 | 32937 |
| 9522.1.1 | KAL | 52 | 32747 | 453 | - | - | 37258 | 36404 | 37748 | 36223 | 38865 |
| 11435.1.1 | KAL | 83 | 36445 | 408 | - | - | 41433 | 41146 | 41782 | 40799 | 42026 |
| 11436.1.2 | KAL | 96 | 34392 | 330 | - | - | 39559 | 39228 | 39880 | 38757 | 40458 |
| 11437.1.1 | KAL | 120 | 36032 | 392 | - | - | 41097 | 40737 | 41419 | 40404 | 41843 |
| 9784.1.1 | KAL | 144 | 37362 | 324 | - | - | 42009 | 41860 | 42204 | 41537 | 42314 |
| 9528.1.1 | KAL | 146 | 43025 | 679 | - | - | 45487 | 44898 | 45974 | 44496 | 46746 |
| 9785.1.1 | KAL | 148 | >42453 | - | - | - | infinite age | - | - | - | - |
| 9529.1.1 | KAL | 154 | >43059 | - | - | - | infinite age | - | - | - | - |
| 9529.1.1 | KAL | 154 | >42943 | - | - | - | infinite age | - | - | - | - |
| 9530.1.1 | KAL | 166 | >43076 | - | - | - | infinite age | - | - | - | - |
| 9531.1.1 | KAL | 178 | >50047 | - | - | - | infinite age | - | - | - | - |

**Remarks: Radiocarbon calibration program CALIB 8.20; InterCal20 age calibration curve**

**Supplementary Table 2**

Tie points used for the development of the age model. Different color codes are used to highlight type of tie points, i.e., AMS$^{14}$C from planktic foraminifers, Relative paleointensity (RPI) values, geomagnetic excursions, and $^{230}Th_{ex}$ and $^{231}Pa_{ex}$ data (for further explanations see Figs. 2 and 3 as well as Supplementary Fig. 8). Ages of geomagnetic excursions according to refs. 18 and 66. "Code" gives tie point numbers (in red RPI, in black geomagnetic excursions) used in Figure 2. (a) Proposed age model used in this study; (b) alternative age model.

**(a) Proposed age model**

| Depth (cm) | Age (ka) | Age Fix Point | Code | Coring Gear |
|---|---|---|---|---|
| 0,5 | 0,22 | AMS14C (planktic foraminifers) | | PS115/2-2-1 GKG |
| 3,0 | 1,14 | AMS14C (planktic foraminifers) | | PS115/2-2-1 GKG |
| 5,0 | 3,23 | AMS14C (planktic foraminifers) | | PS115/2-2-1 GKG |
| 10,0 | 8 | RPI VADM Channell-09 | 1 | PS115/2-2-1 GKG |
| 31,0 | 21 | RPI VADM Channell-09 | 2 | PS115/2-2-1 GKG |
| 78,6 | 32 | Mono Lake | 1 | PS115/2_2-2 KAL |
| 126,7 | 41 | Laschamp | 2 | PS115/2_2-2 KAL |
| 196,7 | 60 | RPI VADM Channell-09 | 3 | PS115/2_2-2 KAL |
| 220,0 | 67 | RPI VADM Channell-09 | 4 | PS115/2_2-2 KAL |
| 259,1 | 92 | RPI VADM Channell-09 | 5 | PS115/2_2-2 KAL |
| 293,4 | 114 | Blake | 3 | PS115/2_2-2 KAL |
| 305,0 | 120 | 231Paex extinction | | PS115/2_2-2 KAL |
| 314,4 | 125 | RPI VADM Channell-09 | 6 | PS115/2_2-2 KAL |
| 332,0 | 130 | 230Thex MIS 5/6 | | PS115/2_2-2 KAL |
| 372,0 | 168 | RPI VADM Channell-09 | 7 | PS115/2_2-2 KAL |
| 457,0 | 188 | Iceland Basin | 4 | PS115/2_2-2 KAL |
| 477,4 | 212 | Pringle Falls | 5 | PS115/2_2-2 KAL |
| 501,1 | 231 | RPI VADM Channell-09 | 8 | PS115/2_2-2 KAL |
| 508,0 | 245 | RPI VADM Channell-09 | 9 | PS115/2_2-2 KAL |
| 535,0 | 268 | RPI VADM Channell-09 | 10 | PS115/2_2-2 KAL |
| 544,0 | 280 | 230Thex MIS 9/8 | | PS115/2_2-2 KAL |
| 573,0 | 286 | Portuguise Orphan | 6 | PS115/2_2-2 KAL |
| 590,0 | 290 | 230Thex extinction | | PS115/2_2-2 KAL |
| 620 | 300 | RPI VADM Channell-09 | 11 | PS115/2_2-2 KAL |
| 629,6 | 310 | RPI VADM Channell-09 | 12 | PS115/2_2-2 KAL |
| 641 | 335 | RPI VADM Channell-09 | 13 | PS115/2_2-2 KAL |
| 691,7 | 347 | RPI VADM Channell-09 | 14 | PS115/2_2-2 KAL |
| 705,5 | 363 | RPI VADM Channell-09 | 15 | PS115/2_2-2 KAL |
| 719,4 | 394 | RPI VADM Channell-09 | 16 | PS115/2_2-2 KAL |
| 726,3 | 409 | Bermuda | 7 | PS115/2_2-2 KAL |
| 756,3 | 426 | RPI VADM Channell-09 | 17 | PS115/2_2-2 KAL |
| 764,0 | 430 | extrapolation | | PS115/2_2-2 KAL |

**(b) Alternative age model**

| Depth (cm) | Age (ka) | Age Fix Point | Code | Coring Gear |
|---|---|---|---|---|
| 0,5 | 0,22 | AMS14C (planktic foraminifers) | | PS115/2-2-1 GKG |
| 3,0 | 1,14 | AMS14C (planktic foraminifers) | | PS115/2-2-1 GKG |
| 5,0 | 3,23 | AMS14C (planktic foraminifers) | | PS115/2-2-1 GKG |
| 10,0 | 8 | RPI VADM Channell-09 | 1 | PS115/2-2-1 GKG |
| 31,0 | 21 | RPI VADM Channell-09 | 2 | PS115/2-2-1 GKG |
| 78,6 | 32 | Mono Lake | 1 | PS115/2_2-2 KAL |
| 126,7 | 41 | Laschamp | 2 | PS115/2_2-2 KAL |
| 196,7 | 60 | RPI VADM Channell-09 | 3 | PS115/2_2-2 KAL |
| 220,0 | 67 | RPI VADM Channell-09 | 4 | PS115/2_2-2 KAL |
| 259,1 | 92 | RPI VADM Channell-09 | 5 | PS115/2_2-2 KAL |
| 293,4 | 114 | Blake | 3 | PS115/2_2-2 KAL |
| 305,0 | 120 | 231Paex extinction | | PS115/2_2-2 KAL |
| 314,4 | 125 | RPI VADM Channell-09 | 6 | PS115/2_2-2 KAL |
| 332,0 | 130 | 230Thex MIS 5/6 | | PS115/2_2-2 KAL |
| 372,0 | 168 | RPI VADM Channell-09 | 7 | PS115/2_2-2 KAL |
| 373,0 | 170 | Hiatus | | PS115/2_2-2 KAL |
| 373,0 | 190 | Hiatus | | PS115/2_2-2 KAL |
| 434,0 | 205 | RPI VADM Channell-09 | | PS115/2_2-2 KAL |
| 477,4 | 212 | Pringle Falls | 5 | PS115/2_2-2 KAL |
| 501,1 | 231 | RPI VADM Channell-09 | 8 | PS115/2_2-2 KAL |
| 508,0 | 245 | RPI VADM Channell-09 | 9 | PS115/2_2-2 KAL |
| 535,0 | 268 | RPI VADM Channell-09 | 10 | PS115/2_2-2 KAL |
| 544,0 | 280 | 230Thex MIS 9/8 | | PS115/2_2-2 KAL |
| 573,0 | 286 | Portuguise Orphan | 6 | PS115/2_2-2 KAL |
| 590,0 | 290 | 230Thex extinction | | PS115/2_2-2 KAL |
| 620 | 300 | RPI VADM Channell-09 | 11 | PS115/2_2-2 KAL |
| 629,6 | 310 | RPI VADM Channell-09 | 12 | PS115/2_2-2 KAL |
| 641 | 335 | RPI VADM Channell-09 | 13 | PS115/2_2-2 KAL |
| 691,7 | 347 | RPI VADM Channell-09 | 14 | PS115/2_2-2 KAL |
| 705,5 | 363 | RPI VADM Channell-09 | 15 | PS115/2_2-2 KAL |
| 719,4 | 394 | RPI VADM Channell-09 | 16 | PS115/2_2-2 KAL |
| 726,3 | 409 | Bermuda | 7 | PS115/2_2-2 KAL |
| 756,3 | 426 | RPI VADM Channell-09 | 17 | PS115/2_2-2 KAL |
| 764,0 | 430 | extrapolation | | PS115/2_2-2 KAL |

**Supplementary Table 3**

Summary of characteristics of the dark gray organic-carbon (OC)-rich intervals.
Sed rate = Sedimentation rate; DD = Dry density; OC AccR = Organic carbon accumulation rate

| PS115/2-2 OC Interval | | Depth | Age | MIS | Thickness | Duration | Sed rate | Mean TOC | Mean DD | OC AccR |
|---|---|---|---|---|---|---|---|---|---|---|
| | | (cm) | (ka) | | (cm) | (ky) | (cm/ky) | (%) | (g/cm3) | (mg/cm2/ky) |
| **OC-1** | Top | 178 | 54,9 | MIS 4/3 | 20 | 5,5 | 3,7 | 1,58 | 0,81 | 47 |
| | Bottom | 198 | 60,4 | | | | | | | |
| **OC-2** | Top | 226 | 70,8 | Late MIS 5 (b?) | 52 | 33,3 | 1,6 | 1,52 | 0,81 | 20 |
| | Bottom | 278 | 104,1 | | | | | | | |
| **OC-3** | Top | 326 | 128,3 | MIS 6 | 46 | 39,7 | 1,5 | 1,64 | 0,8 | 20 |
| | Bottom | 372 | 168,0 | | | | | | | |
| **OC-4** | Top | 428 | 181,2 | early MIS 6 | 10 | 2,3 | 4,3 | 0,95 | 0,86 | 35 |
| | Bottom | 438 | 183,5 | | | | | | | |
| **OC-5** | Top | 504 | 236,9 | MIS 8(/7) | 36 | 37,8 | 1,2 | 1,28 | 0,96 | 15 |
| | Bottom | 540 | 274,7 | | | | | | | |
| **OC-6** | Top | 640 | 332,8 | MIS 10 | 68 | 32,9 | 3,2 | 1,54 | 0,93 | 46 |
| | Bottom | 708 | 365,5 | | | | | | | |
| **OC-7** | Top | 744 | 419,0 | MIS 12/11 | 14 | 7,9 | 1,8 | 1,51 | 0,73 | 20 |
| | Bottom | 758 | 426,9 | | | | | | | |

## Supplementary Table 4

Summary of the biomarker concentrations (in ng/gSediment) determined in samples from Core PS115/2-2-2.

| Depth (cm) | Age (ka) | TOC (%) | IP25 (ng/gSed) | HBI II (ng/gSed) | HBI III (Z) (ng/gSed) | Brassicasterol (ng/gSed) | Dinosterol (ng/gSed) | Campesterol (ng/gSed) | Sitosterol (ng/gSed) | Short-chain n-alk (ng/gSed) | Long-chain n-alk (ng/gSed) | Long/Short n-alkanes | DGD (ng/gSed) | Pristan/Phytan ratio | CPI | OC Intervals |
|---|---|---|---|---|---|---|---|---|---|---|---|---|---|---|---|---|
| 0,5 | 0,221 | 0,43 | 0,248 | 247,612 | 0,000 | 0,537 | 0,488 | 0,752 | 7,274 | 42,101 | 2467,616 | 59 | 22,483 | 3,3 | 6,9 | |
| 15 | 11,095 | 0,38 | 0,000 | 0,000 | 0,000 | 0,648 | 1,827 | 0,261 | 4,687 | 29,726 | 1584,147 | 53 | 18,885 | 2,4 | 6,2 | |
| 25 | 17,286 | 0,37 | 0,000 | 0,000 | 0,000 | 0,000 | 0,505 | 0,460 | 3,439 | 22,119 | 1038,721 | 47 | 13,313 | 3,5 | 6,2 | |
| 35 | 21,924 | 0,27 | 0,000 | 0,000 | 0,000 | 0,000 | 0,929 | 0,000 | 3,025 | 24,118 | 2541,567 | 105 | 13,829 | 2,4 | 4,5 | |
| 45 | 24,235 | 0,24 | 0,000 | 0,000 | 0,000 | 0,514 | 0,000 | 0,082 | 1,187 | 30,403 | 4546,981 | 150 | 16,452 | 3,2 | 4,7 | |
| 55 | 26,546 | 0,23 | 0,000 | 0,000 | 0,000 | 0,000 | 0,000 | 0,000 | 0,000 | 14,335 | 4945,149 | 345 | 14,224 | | 4,6 | |
| 65 | 28,857 | 0,23 | 0,000 | 0,000 | 0,000 | 0,435 | 0,522 | 0,138 | 1,767 | 19,038 | 2004,510 | 105 | 16,686 | 2,9 | 6,0 | |
| 75 | 31,168 | 0,22 | 0,000 | 0,000 | 0,000 | 0,224 | 0,000 | 0,113 | 0,404 | 13,645 | 1040,825 | 76 | 6,532 | 0,9 | 4,9 | |
| 85 | 33,198 | 0,19 | 0,000 | 0,000 | 0,000 | 0,210 | 0,000 | 0,000 | 0,691 | 13,513 | 1331,844 | 99 | 10,850 | 2,1 | 3,8 | |
| 95 | 35,069 | 0,22 | 0,000 | 0,000 | 0,000 | 0,000 | 0,000 | 0,000 | 0,361 | 10,576 | 925,741 | 88 | 8,782 | 3,2 | 4,8 | |
| 105 | 36,940 | 0,18 | 0,000 | 0,000 | 0,000 | 0,000 | 0,000 | 0,000 | 0,000 | 8,832 | 1146,463 | 130 | 9,991 | 2,5 | 6,0 | |
| 115 | 38,811 | 0,17 | 0,000 | 0,000 | 0,000 | 0,639 | 0,306 | 0,000 | 0,715 | 5,427 | 1253,978 | 231 | 10,642 | 3,5 | 3,3 | |
| 125 | 40,682 | 0,17 | 0,000 | 0,000 | 0,000 | 0,580 | 0,114 | 0,033 | 0,433 | 6,022 | 1381,402 | 229 | 16,209 | 1,1 | 5,5 | |
| 135 | 43,253 | 0,16 | 0,000 | 0,000 | 0,000 | 0,757 | 0,000 | 0,084 | 1,200 | 8,878 | 1647,743 | 186 | 24,454 | 3,4 | 6,0 | |
| 145 | 45,967 | 0,40 | 0,000 | 0,000 | 0,000 | 0,099 | 0,000 | 0,112 | 0,355 | 20,847 | 295,383 | 14 | 5,977 | 1,5 | 3,2 | |
| 155 | 48,681 | 0,37 | 0,000 | 0,000 | 0,000 | 0,000 | 0,307 | 0,000 | 0,326 | 12,555 | 230,002 | 18 | 3,570 | 2,1 | 2,9 | |
| 165 | 51,396 | 0,46 | 0,000 | 0,000 | 0,000 | 0,000 | 0,000 | 0,000 | 0,058 | 15,682 | 314,214 | 20 | 2,683 | 2,5 | 3,0 | |
| 175 | 54,110 | 0,70 | 0,000 | 0,000 | 0,000 | 0,588 | 0,995 | 1,191 | 5,362 | 65,945 | 820,230 | 12 | 16,678 | 1,7 | 3,2 | |
| 185 | 56,824 | 2,19 | 0,391 | 0,828 | 0,789 | 9,374 | 16,562 | 13,259 | 86,589 | 219,137 | 1257,980 | 6 | 157,014 | 1,3 | 3,4 | OC-1 |
| 195 | 59,539 | 1,99 | 0,339 | 0,783 | 0,574 | 9,633 | 19,160 | 8,319 | 82,970 | 185,991 | 1060,507 | 6 | 120,626 | 1,4 | 3,3 | OC-1 |
| 205 | 62,494 | 0,35 | 0,000 | 0,000 | 0,000 | 2,420 | 2,212 | 1,181 | 9,442 | 55,382 | 1881,826 | 34 | 49,698 | 2,2 | 4,7 | |
| 215 | 65,498 | 0,62 | 0,000 | 0,000 | 0,000 | 1,085 | 0,733 | 0,136 | 7,017 | 36,166 | 273,208 | 8 | 16,800 | 1,7 | 3,2 | |
| 225 | 70,197 | 0,99 | 0,000 | 0,029 | 0,025 | 1,592 | 2,006 | 3,886 | 18,881 | 94,833 | 723,225 | 8 | 49,801 | 1,7 | 3,4 | |
| 235 | 76,591 | 2,41 | 0,248 | 0,665 | 0,544 | 14,060 | 15,754 | 13,767 | 103,140 | 183,624 | 1135,297 | 6 | 156,755 | 1,2 | 3,6 | OC-2 |
| 245 | 82,985 | 2,26 | 0,181 | 0,689 | 0,639 | 19,188 | 18,015 | 17,166 | 133,021 | 162,781 | 1027,470 | 6 | 136,349 | 1,5 | 3,5 | OC-2 |
| 255 | 89,379 | 1,86 | 0,384 | 1,049 | 0,959 | 15,566 | 20,931 | 10,380 | 92,336 | 150,634 | 1081,558 | 7 | 163,468 | 1,2 | 3,5 | OC-2 |
| 265 | 95,784 | 1,67 | 0,209 | 0,615 | 0,890 | 20,049 | 17,470 | 12,911 | 122,684 | 156,418 | 884,458 | 6 | 125,914 | 1,4 | 3,5 | OC-2 |
| 275 | 102,198 | 1,62 | 0,226 | 0,670 | 0,897 | 23,689 | 16,159 | 18,126 | 116,053 | 121,574 | 955,593 | 8 | 108,212 | 1,2 | 3,5 | OC-2 |
| 285 | 108,612 | 0,28 | 0,000 | 0,000 | 0,000 | 1,307 | 0,388 | 0,298 | 3,073 | 75,201 | 1428,800 | 19 | 21,721 | 3,0 | 4,0 | |
| 295 | 114,838 | 0,31 | 0,000 | 0,000 | 0,000 | 0,730 | 0,296 | 0,093 | 1,513 | 21,522 | 655,374 | 30 | 11,355 | 2,1 | 4,2 | |
| 305 | 120,076 | 0,42 | 0,000 | 0,000 | 0,000 | 0,580 | 1,446 | 0,535 | 4,988 | 23,254 | 415,351 | 18 | 14,186 | 1,4 | 3,1 | |
| 315 | 125,451 | 0,75 | 0,000 | 0,046 | 0,000 | 0,820 | 2,316 | 2,578 | 14,396 | 44,098 | 556,533 | 13 | 20,588 | 1,7 | 3,4 | |
| 325 | 128,011 | 1,66 | 0,069 | 0,247 | 0,077 | 7,538 | 14,849 | 9,979 | 81,268 | 151,803 | 2469,976 | 16 | 123,545 | 1,7 | 3,8 | OC-3 |
| 335 | 132,850 | 2,06 | 0,353 | 1,280 | 0,641 | 23,574 | 25,513 | 24,463 | 227,954 | 154,705 | 1364,857 | 9 | 178,015 | 1,2 | 3,8 | OC-3 |
| 345 | 142,350 | 2,12 | 0,232 | 1,171 | 0,483 | 18,607 | 23,300 | 15,089 | 179,365 | 165,368 | 1459,077 | 9 | 181,677 | 1,3 | 3,7 | OC-3 |
| 355 | 151,850 | 1,38 | 0,190 | 0,580 | 0,338 | 12,123 | 19,972 | 13,287 | 138,822 | 91,312 | 857,477 | 9 | 156,857 | 1,4 | 3,8 | OC-3 |
| 365 | 161,350 | 1,64 | 0,325 | 0,882 | 0,377 | 15,363 | 21,463 | 23,461 | 219,989 | 111,493 | 1363,147 | 12 | 186,274 | 1,4 | 3,9 | OC-3 |
| 375 | 168,706 | 0,32 | 0,000 | 0,050 | 0,000 | 3,215 | 3,904 | 1,260 | 11,345 | 72,358 | 2010,999 | 28 | 69,406 | 2,1 | 2,3 | |
| 385 | 171,059 | 0,25 | 0,000 | 0,000 | 0,000 | 1,347 | 1,948 | 0,074 | 4,635 | 11,826 | 953,498 | 81 | 20,375 | 2,4 | 6,0 | |
| 395 | 173,412 | 0,26 | 0,000 | 0,000 | 0,000 | 1,170 | 1,166 | 0,026 | 3,996 | 6,928 | 1249,342 | 180 | 19,273 | 2,5 | 6,8 | |
| 405 | 175,765 | 0,22 | 0,000 | 0,000 | 0,000 | 0,504 | 2,759 | 0,072 | 1,942 | 4,444 | 728,859 | 164 | 16,549 | 2,0 | 4,5 | |
| 415 | 178,118 | 0,43 | 0,000 | 0,000 | 0,000 | 0,373 | 1,081 | 0,345 | 4,961 | 21,018 | 342,168 | 16 | 13,384 | 2,2 | 3,3 | |
| 425 | 180,471 | 0,79 | 0,000 | 0,039 | 0,000 | 2,104 | 1,994 | 1,560 | 18,351 | 61,962 | 728,031 | 12 | 41,286 | 1,9 | 3,7 | |
| 435 | 182,824 | 1,37 | 0,116 | 0,176 | 0,096 | 5,415 | 11,258 | 4,363 | 31,070 | 117,061 | 1443,086 | 12 | 62,393 | 1,2 | 3,5 | OC-4 |
| 445 | 185,176 | 0,30 | 0,000 | 0,000 | 0,000 | 0,467 | 3,280 | 0,918 | 7,247 | 8,835 | 1034,218 | 117 | 17,223 | 3,7 | 6,4 | |
| 455 | 187,529 | 0,28 | 0,000 | 0,000 | 0,000 | 0,322 | 5,023 | 1,100 | 11,123 | 13,305 | 1507,821 | 113 | 26,573 | 2,2 | 6,1 | |
| 465 | 197,412 | 0,26 | 0,000 | 0,000 | 0,000 | 0,698 | 1,377 | 0,645 | 7,255 | 14,024 | 1361,298 | 97 | 23,770 | 2,8 | 1,6 | |
| 475 | 209,176 | 0,31 | 0,000 | 0,000 | 0,000 | 0,333 | 2,065 | 0,408 | 2,725 | 17,982 | 1784,070 | 99 | 19,901 | 2,7 | 5,6 | |
| 485 | 218,093 | 0,53 | 0,000 | 0,000 | 0,000 | 0,112 | 0,216 | 0,389 | 6,895 | 50,619 | 265,544 | 5 | 13,887 | 2,1 | 3,2 | |
| 495 | 226,110 | 0,63 | 0,000 | 0,000 | 0,000 | 1,039 | 2,948 | 2,699 | 40,621 | 38,685 | 439,383 | 11 | 21,589 | 1,9 | 3,2 | |
| 505 | 238,913 | 1,51 | 0,146 | 0,432 | 0,291 | 12,422 | 15,308 | 9,537 | 83,026 | 100,441 | 1070,612 | 11 | 145,254 | 1,2 | 3,8 | OC-5 |
| 515 | 250,963 | 1,62 | 0,222 | 0,710 | 0,550 | 17,382 | 15,635 | 13,495 | 116,845 | 103,364 | 980,280 | 9 | 141,064 | 1,4 | 3,8 | OC-5 |
| 525 | 259,481 | 1,63 | 0,573 | 1,076 | 0,787 | 14,465 | 23,663 | 12,747 | 123,228 | 106,040 | 1046,398 | 10 | 64,291 | 1,2 | 3,7 | OC-5 |
| 535 | 268,000 | 1,70 | 0,210 | 0,622 | 0,444 | 18,988 | 19,756 | 21,219 | 139,787 | 110,052 | 1120,615 | 10 | 132,870 | 1,2 | 3,8 | OC-5 |
| 545 | 272,737 | 0,34 | 0,070 | 0,081 | 0,000 | 0,951 | 10,267 | 0,917 | 7,312 | 40,155 | 1320,791 | 33 | 60,191 | 1,7 | 5,3 | |
| 555 | 277,474 | 0,25 | 0,000 | 0,070 | 0,000 | 0,218 | 2,323 | 1,395 | 8,839 | 19,668 | 598,683 | 30 | 29,003 | 0,7 | 3,6 | |
| 565 | 282,211 | 0,39 | 0,000 | 0,035 | 0,000 | 3,126 | 3,354 | 2,057 | 12,505 | 38,702 | 1021,177 | 26 | 32,763 | 1,7 | 4,1 | |
| 575 | 286,596 | 0,25 | 0,000 | 0,000 | 0,000 | 0,444 | 0,601 | 0,197 | 4,898 | 10,304 | 955,830 | 93 | 16,682 | 2,4 | 5,9 | |
| 585 | 289,574 | 0,22 | 0,000 | 0,000 | 0,000 | 0,067 | 0,502 | 0,134 | 0,264 | 11,433 | 625,421 | 55 | 10,049 | 2,3 | 5,0 | |
| 595 | 292,553 | 0,20 | 0,000 | 0,000 | 0,000 | 0,000 | 1,249 | 0,298 | 0,234 | 5,168 | 665,266 | 129 | 16,948 | 1,5 | 5,2 | |
| 603 | 294,936 | 0,32 | 0,000 | 0,000 | 0,000 | 0,060 | 0,923 | 0,256 | 4,388 | 10,857 | 383,208 | 35 | 16,536 | 2,1 | 4,0 | |
| 605 | 295,532 | 0,48 | 0,000 | 0,000 | 0,000 | 0,383 | 0,089 | 0,854 | 6,654 | 16,013 | 656,460 | 41 | 21,933 | 1,8 | 3,8 | |
| 615 | 298,511 | 0,40 | 0,000 | 0,000 | 0,000 | 0,262 | 0,541 | 0,419 | 10,206 | 15,740 | 830,952 | 53 | 13,420 | 2,3 | 2,8 | |
| 625 | 305,208 | 0,32 | 0,000 | 0,000 | 0,000 | 0,536 | 0,000 | 0,100 | 4,094 | 6,869 | 687,639 | 100 | 13,376 | 1,3 | 3,8 | |
| 635 | 321,842 | 0,46 | 0,000 | 0,000 | 0,000 | 0,269 | 0,060 | 0,108 | 3,929 | 14,099 | 711,406 | 50 | 16,419 | 1,5 | 3,8 | |
| 645 | 335,947 | 2,10 | 0,498 | 2,035 | 0,219 | 15,606 | 35,391 | 37,665 | 485,514 | 132,498 | 1860,757 | 14 | 284,276 | 1,4 | 4,3 | OC-6 |
| 655 | 338,314 | 1,97 | 1,055 | 3,285 | 0,448 | 13,016 | 34,991 | 28,584 | 409,112 | 175,093 | 2608,582 | 15 | 492,736 | 1,2 | 4,4 | OC-6 |
| 665 | 340,680 | 1,56 | 0,502 | 1,687 | 0,211 | 11,776 | 29,789 | 30,849 | 400,627 | 100,665 | 2258,667 | 22 | 282,529 | 1,8 | 4,3 | OC-6 |
| 675 | 343,047 | 2,29 | 0,373 | 1,364 | 0,203 | 14,570 | 19,485 | 21,318 | 299,458 | 99,963 | 884,556 | 9 | 187,618 | 1,3 | 4,1 | OC-6 |
| 685 | 345,414 | 0,72 | 0,083 | 0,511 | 0,060 | 1,438 | 9,798 | 4,866 | 77,085 | 36,253 | 595,388 | 16 | 117,437 | 1,6 | 4,2 | OC-6 |
| 695 | 350,826 | 1,95 | 0,393 | 1,183 | 0,200 | 12,325 | 33,904 | 33,795 | 326,995 | 108,723 | 1165,774 | 11 | 204,143 | 1,3 | 4,1 | OC-6 |
| 705 | 362,420 | 1,65 | 0,473 | 1,500 | 0,332 | 9,910 | 14,397 | 20,708 | 194,619 | 106,747 | 1356,801 | 13 | 263,548 | 1,1 | 3,9 | OC-6 |
| 713 | 373,243 | 0,32 | 0,000 | 0,094 | 0,000 | 0,895 | 3,743 | 1,393 | 7,452 | 36,782 | 1732,033 | 47 | 60,711 | 2,0 | 4,0 | |
| 715 | 379,730 | 0,35 | 0,000 | 0,000 | 0,000 | 0,709 | 0,171 | 0,349 | 6,133 | 28,702 | 967,175 | 34 | 35,159 | 1,8 | 4,5 | |
| 725 | 406,174 | 0,45 | 0,000 | 0,000 | 0,000 | 1,156 | 1,027 | 1,321 | 6,826 | 25,451 | 405,201 | 16 | 11,975 | 1,6 | 3,1 | |
| 735 | 413,930 | 0,56 | 0,000 | 0,000 | 0,000 | 0,423 | 0,662 | 0,864 | 10,294 | 29,427 | 436,591 | 15 | 15,698 | 1,7 | 3,4 | |
| 745 | 419,597 | 1,52 | 0,293 | 0,441 | 0,132 | 7,538 | 15,009 | 16,791 | 122,034 | 112,097 | 1245,203 | 11 | 116,390 | 1,4 | 3,8 | OC-7 |
| 755 | 425,263 | 2,13 | 1,365 | 2,943 | 1,012 | 24,607 | 39,286 | 40,458 | 414,881 | 206,512 | 1995,931 | 10 | 84,776 | 1,1 | 3,9 | OC-7 |
| 765 | 430,563 | 0,47 | 0,000 | 0,000 | 0,000 | 0,708 | 0,058 | 1,138 | 10,112 | 35,546 | 395,002 | 11 | 27,144 | 1,8 | 3,4 | |

## (7) References Supplement Information


1. Stein, R. (Ed.). Expedition PS115/2 of the Research Vessel POLARSTERN to the Arctic Ocean in 2018. *Reps. Pol. Mar. Res.* **728**, https://epic.awi.de/id/eprint/49226/1/BzPM_0728_2019.pdf (2019).
2. Stein, R., St. John, K., and Everest, J. IODP Expedition 377 Scientific Prospectus: Arctic Ocean Paleoceanography (ArcOP); https://doi.org/10.14379/iodp.sp.377.2021 (2021).
3. Jakobsson, M. et al. The International Bathymetric Chart of the Arctic Ocean (IBCAO) Version 3.0. *Geophys. Res. Lett.* **39**, L12609 (2012).
4. Stein, R. Documentation of sediment core PS115/2-2-2 (X-Ray photographs). AWI Polarstern Core Repository, PANGAEA, https://doi.org/10.1594/PANGAEA.896181 (2018).
5. Sassenroth, C. A 500ka Deep-Sea Record of the Central Arctic Ocean: Paleoclimate Reconstruction from Sedimentological and Geochemical Data. Unpubl. Master Thesis, Universities of Hamburg and St. Petersburg. 99 pp. (2019).
6. Rachor, E. (Ed.). Scientific cruise report of the Arctic Expedition ARK-XI/1 of RV "Polarstern" in 1995. *Rep. Pol. Res.* **226**, 157 pp.; https://epic.awi.de/id/eprint/26404/ (1997).
7. Stein, R., Behrends, M., & Spielhagen, R.F. Lithostratigraphy and sediment characteristics. *Rep. Pol. Res.* **226**, 143-153; https://epic.awi.de/id/eprint/26404/ (1997).
8. Stein, R. (Ed.), 2015. The Expedition PS87 of the Research Vessel *Polarstern* to the Arctic Ocean in 2014, *Reps. Pol. Mar. Res.* **688** (http://epic.awi.de/37728/1/BzPM_0688_2015.pdf) (2015).
9. Stein, R., Fahl, K., Gierz, P., Niessen, F., & Lohmann, G. Arctic Ocean sea ice cover during the penultimate glacial and the last interglacial. *Nat. Comm.* **8**, 373 (2017).
10. West, G., Alexanderson, H., Jakobsson, M. & O'Regan, M. Optically stimulated luminescence dating supports pre-Eemian age for glacial ice on the Lomonosov Ridge off the East Siberian continental shelf. *Quat. Sci. Rev.* **267**, 107082 (2021).
11. Purcell, K., Hillaire-Marcel, C., de Vernal, A., Ghaleb, B. & Stein, R. Potential and limitation of $^{230}$Th-excess as a chronostratigraphic tool for late Quaternary Arctic Ocean sediment studies: An example from the Southern Lomonosov Ridge. *Mar. Geol.* **448**, 106802 (2022).
12. Stein, R., et al. Accumulation of particulate organic carbon at the Eurasian continental margin during late Quaternary times: Controlling mechanisms and paleoenvironmental significance. *Glob. Plan. Change* **31**/1-4, 87-102 (2001).
13. Kosterov, A. Low-temperature magnetization and AC susceptibility of magnetite: effect of thermomagnetic history. *Geophys. J. Int.* **154**, 58–71, https://doi.org/10.1046/j.1365-246X.2003.01938.x (2003).
14. Özdemir, Ö. & Dunlop, D.J. Hallmarks of maghemitization in low-temperature remanence cycling of partially oxidized magnetite nanoparticles. *J. Geophys. Res.* **115**, B02101 (2010).
15. Verwey, E. J. W. Electronic conduction of magnetite (Fe3O4) and its transition point at low temperatures. *Nature* **144**, 327–328, doi:10.1038/144327b0 (1939).
16. Moskowitz, B.M., Jackson, M., & Kissel, C. Low-temperature magnetic behavior of titanomagnetites. *Earth Planet. Sci. Lett.* **157**, https://doi.org/10.1016/S0012-821X(98)00033-8 (1998).
17. Frederichs, T. von Dobeneck, T., Bleil, U., & Dekkers, M.J. Towards the identification of siderite, rhodochrosite, and vivianite in sediments by their low-temperature magnetic properties. *Phys. Chem. Earth* **28**, https://doi.org/10.1016/S1474-7065(03)00121-9 (2003).
18. Channell, J.E.T., Singer, B.S. & Jicha, B.R., 2020. Timing of Quaternary geomagnetic reversals and excursions in volcanic and sedimentary archives. *Quat. Sci. Rev.* **228** (2020).
19. Schulz, M. & Stattegger, K. Spectrum: spectral analysis of unevenly spaced paleoclimatic time series, *Computers & Geosciences* **23/9**, 929-945 (1997).
20. Channell, J.E.T., Xuan, C. & Hodell, D.A. Stacking paleointensity and oxygen isotope data for the last 1.5 Myrs (PISO 1500). *Earth Planet. Sci. Lett.* **283**, 14-23 (2009).

21. Lisiecki, L. E. & Raymo, M.E. A Pliocene-Pleistocene stack of 57 globally distributed benthic d$^{18}$O records. *Paleoceanography* **20**, PA1003 (2005).
22. Naafs, B.D.A., Blewett, J., & Pancost, R.D. Bacterial diether lipids as a novel proxy to reconstruct past changes in sedimentary oxygenation. *Geochim. Cosmochim. Acta*; https://doi.org/10.1016/ j.gca.2024.07.036 (2024).
23. Didyk, B. M., Simoneit, B. R. T., Brassell, S. C. & Eglinton, G. Organic geochemical indicators of palaeoenvironmental conditions of sedimentation. *Nature* **272**, 216-222 (1978).
24. Ten Haven, H.L., de Leeuw, J.W., Rullkötter, J., & Sinninghe Damsté, J.S. Restricted utility of the pristane/phytane ratio as a palaeoenvironmental indicator. *Nature* **330**, 17 (1987).
25. Bray, E.E., Evans, E.D., 1961. Distribution of n-paraffins as a clue to recognition of source beds. *Geochim. Cosmochim. Acta* **22**, 2–15.
26. Marzi, R., Torkelson, B.E., Olson, R.K., 1993. A revised carbon preference index. *Org. Geochem.* **20**, 1303-1306.
27. Herrera-Herrera, A.V., Leierer, L., Jambrina-Enriquez, M., Connolly, R., Mallo, C., 2020. Evaluating different methods for calculating the Carbon Preference Index (CPI): Implications for palaeoecological and archaeological research. *Org. Geochem*. **146**, 104056 (2020).
28. Möller, P., Alexanderson, H., Funder, S. & Hjort, C. The Taimyr Peninsula and the Severnaya Zemlya archipelago, Arctic Russia: a synthesis of glacial history and palaeo-environmental change during the Last Glacial cycle (MIS 5e-2). *Quat. Sci. Rev.* **107**, 149-181 (2015).
29. Lorenz, H., Männik, P., Gee, D. & Proskurnin, V. Geology of the Severnaya Zemlya Archipelago and the North Kara Terrane in the Russian high Arctic. *Int. Journ. Earth Sci.* **97**, 519-547 (2007).
30. Zhang, X., Pease, V., Omma, J. & Benedictus, A. Provenance of Late Carboniferous to Jurassic sandstones for southern Taimyr, Arctic Russia: A comparison of heavy mineral analysis by optical and QEMSCAN methods. *Sedim. Geol.* **329**, 166-176 (2015).
31. Shishlov, S.B. & Dubkova, K.A. Depositional Environments and Paleogeographic Evolution during the Formation of Upper Paleozoic Terrigenous Rocks in Taimyr. *Lithol. Min. Resources* **56/6**, 509–522 (2021).
32. Rudmin, M., Banerjee, S., Sinkina, E., Ruban, A., Kalinina, N. & Smirnov, P. A study of iron carbonates and clay minerals for understanding the origin of marine ooidal ironstone deposits. *Mar. Petrol. Geol.* **142**, 105777 (2022).
33. Knies, J., Kleiber, H.P., Matthiessen, Müller, C. & Nowaczyk, N. 2001. Marine ice-rafted debris records constrain maximum extent of Saalian and Weichselian ice-sheets along the northern Eurasian margin. *Glob. Planet. Change* **31**, 45-64 (2001).
34. Weiel, D. Paläozeanographische Untersuchungen in der Vilkitsky Straße und östlich von Severnaya Zemlya mit sedimentologischen und geophysikalischen Methoden. Unpubl. diploma thesis, University of Köln, 138 pp., hdl:10013/epic.33306.d001(1997).
35. Kleiber, H.P., Niessen, F. & Weiel, D. The Late Quaternary evolution of the western Laptev Sea continental margin, Arctic Siberia - implications from sub-bottom profiling. *Glob. Planet. Change* **31**, 105-124 (2001).
36. Strobl, C. et al. Determination of depositional Beryllium-10 fluxes in the area of the Laptev Sea and Beryllium-10 concentrations in water samples of high northern latitudes. In: Kassens, H. et al. (Eds.), Land-Ocean Systems in the Siberian Arctic: Dynamics and History. Springer, Berlin, pp. 515-532 (1999).
37. Stein, R., Fahl, K., Niessen, F., & Siebold, M. Late Quaternary Organic Carbon and Biomarker Records from the Laptev Sea Continental Margin: Implications for organic carbon flux and composition. In: Kassens, H., et al. (Eds.), Land-Ocean Systems in the Siberian Arctic: Dynamics and History, Springer-Verlag, Berlin, 635-655 (1999).
38. Müller, C. Rekonstruktion der Paläo-Umweltbedingungen am Laptev-See-Kontinentalrand während der beiden letzten Glazial-/Interglazial-Zyklen anhand sedimentologischer und mineralogischer Untersuchungen. *Rep. Pol. Res.* **328**, 146 pp. hdl:10013/epic.10331.d001 (1999).
39. Matthiessen, J., Knies, J., Nowaczyk, N. & Stein, R. Late Quaternary dinoflagellate cyst ecostratigraphy along the Eurasian Continental Margin (Arctic Ocean): Variability of Atlantic water inflow in the last 150,000 years. *Glob. Plan. Change* **31**/1-4, 65-86 (2001).

40. Stein, R. (Ed.). Scientific Cruise Report of the Arctic Expedition ARK-XX/3 of RV "Polarstern" in 2004: Fram Strait, Yermak Plateau and East Greenland Continental Margin, *Reps. Pol. Mar. Res.* **517**, (doi:https://doi.org/10.2312/BzPM_0517_2005 , hdl:10013/epic.10522 (2005).
41. Fütterer, D. K. (ed.) ARCTIC '91: The expedition ARK-VIII3 of RV "Polarstern" in 1991. *Berichte Polarforsch.* **107**, 1–267; https://doi.org/10.2312/BzP_0107_1992 (1992).
42. Jokat, W. (ed.) The expedition of the research vessel "Polarstern" to the Arctic in 2008 (ARK-XXIII/3). *Berichte Polar- Meeresforsch.* **597**, 1–266; hdl:10013/epic.33317 (2009).
43. Svindland, K.T. & Vorren, T.O. Late Cenozoic sedimentary environments in the Amundsen Basin, Arctic Ocean. *Mar. Geol.* **186**, 541-555 (2002).
44. Winkelmann, D., Schäfer, Ch., Stein, R. & Mackensen, A. Terrigenous events and climate history of the Sophia Basin, Arctic Ocean. *Geochem. Geophys. Geosyst.* **9**, Q07023 (2008)
45. Knies, J., Müller, C., Nowaczyk, N., Vogt, Ch. & Stein, R. A multiproxy approach to reconstruct the environmental changes along the Eurasian continental margin over the last 150 kyr. *Mar. Geol.* **163**, 317-344 (2000).
46. Schubert C. J. Organischer Kohlenstoff in spätquartären Sedimenten des Arktischen Ozeans: Terrigener Eintrag und marine Produktivität. *Reps. Pol. Res.* **177** hdl:10013/epic.10178.d001(1995).
47. Schubert, C. & Stein, R. Deposition of organic carbon in Arctic Ocean sediments: Terrigenous supply vs marine productivity. *Org. Geochem.* **24**, 421-436 (1996).
48. Stein, R. et al. Towards a better (litho-)stratigraphy and reconstruction of Quaternary paleoenvironment in the Amerasian Basin (Arctic Ocean). *Polarforschung* **79**, 97–121; https://epic.awi.de/id/eprint/22435/1/Ste2010b.pdf (2010).
49. Clark, D.L., Whitman, R. R., Morgan, K. A. &Mackey, S. D. Stratigraphy and glacialmarine sediments of the Amerasian Basin, central Arctic Ocean. *Geol. Soc. Am. Spec. Pap.* **181** (1980).
50. Stein, R., Matthiessen, J., & Niessen, F. Re-Coring at Ice Island T3 Site of Key Core FL-224 (Nautilus Basin, Amerasian Arctic): Sediment Characteristics and Stratigraphic Framework. *Polarforschung* **79**, 81-96; https://epic.awi.de/id/eprint/22436/1/Ste2010c.pdf (2010).
51. Schulte-Loh, I. Paläoumweltbedingungen im spätquartären Arktischen Ozean: Rekonstruktion nach sedimentologischen Untersuchungen an Sedimentkernen. Diplomarbeit Universität Bremen (2010).
52. Zou, H. An X-ray diffraction approach: Bulk mineral assemblages as provenance indicator of sediments from the Arctic Ocean. Unpubl. PhD thesis, University of Bremen, 116 pp. (2016)
53. Zou, H. Quantitative X-ray diffraction analysis evaluated by RockJock of sediment core PS72/396-5 [dataset]. *PANGAEA*, https://doi.org/10.1594/PANGAEA.863339 (2016a).
54. Zou, H. Quantitative X-ray diffraction analysis evaluated by RockJock of sediment core PS72/410-3 [dataset]. *PANGAEA*, https://doi.org/10.1594/PANGAEA.863340 (2016b).
55. Zou, H. Quantitative X-ray diffraction analysis evaluated by RockJock of sediment core PS72/422-5 [dataset]. *PANGAEA*, https://doi.org/10.1594/PANGAEA.863341 (2016c).
56. Hodell, D.A., Channell, J.E., Curtis, J.H., Romero, O.E. & Röhl, U. Onset of ''Hudson Strait'' Heinrich events in the eastern North Atlantic at the end of the middle Pleistocene transition (640 ka)? *Paleoceanography* **23**, PA4218 (2008).
57. Stein, R., Hefter, J., Grützner, J., Voelker, A. & Naafs, B.D.A.Variability of surface-water characteteristics and Heinrich Events in the Pleistocene mid-latitude North Atlantic Ocean: Biomarker and XRD records from IODP Site U1313 (MIS 16-9). *Paleoceanography* **24**, PA2203, doi:10.1029/2008PA001639.
58. Elkina, D. Paleomagnetic and rock magnetic data of sediment core PS72/396-5 [dataset bundled publication]. *PANGAEA*, https://doi.org/10.1594/PANGAEA.949227 (2022)
59. Elkina, D., Piskarev, A.L. & Bezumov, D.V. Sedimentation in the Central Arctic Submarine Elevations: Results of Comprehensive Analysis of Paleomagnetic and Seismoacoustic Data. *Geotectonics* **57**, S100-S111 (2023).
60. Jones, G.A. The central Arctic Ocean sediment record: current progress in moving from a litho- to a chronostratigraphy. *Pol. Res.* **5**, 309-311 (1987).
61. Geibert, W., Matthiessen, J., Stimac, I., Wollenburg, J. & Stein, R. Glacial episodes of a freshwater Arctic Ocean covered by a thick ice shelf. *Nature* **590**, 97-102 (2021).

62. Mollenhauer, G., Grotheer, H., Gentz, T., Bonk, E., & Hefter, J. Standard operation procedures and performance of the MICADAS radiocarbon laboratory at Alfred Wegener Institute (AWI), Germany. *Nucl. Instrum. Meth. Phys. Res.* **B 496**, https://doi.org/10.1016/j.nimb.2021.03.016 (2021).
63. Heaton, T. J. et al. Marine20 - The Marine Radiocarbon Age Calibration Curve (0-55,000 cal BP). *Radiocarbon* **62**, https://doi.org/10.1017/RDC.2020.68 (2020).
64. Nicolas, A. et al. Precise dating of deglacial Laptev Sea sediments via $^{14}C$ and authigenic $^{10}Be/^{9}Be$ – assessing local $^{14}C$ reservoir ages. *EGUsphere*, https://doi.org/10.5194/egusphere-2024-1992 (2024).
65. Reimers, P.J., et al. The IntCal20 Northern Hemisphere radiocarbon age calibration curve (0 – 55 Cal kBP). *Radiocarbon* **62**, 725-757 (2020).
66. Ogg, J.G. in *Geologic Time Scale 2020* (eds Gradstein, F. M., Ogg, J. G., Schmitz, M. D., & Ogg, G. M.) 159-192 (Elsevier, 2020) https://doi.org/10.1016/B978-0-12-824360-2.00005-X (2020).
